# Small molecule inhibitors of the mitochondrial ClpXP protease possess cytostatic potential and re-sensitize chemo-resistant cancers

**DOI:** 10.1038/s41598-021-90801-7

**Published:** 2021-05-27

**Authors:** Martina Meßner, Melanie M. Mandl, Mathias W. Hackl, Till Reinhardt, Maximilian A. Ardelt, Karolina Szczepanowska, Julian E. Frädrich, Jens Waschke, Irmela Jeremias, Anja Fux, Matthias Stahl, Angelika M. Vollmar, Stephan A. Sieber, Johanna Pachmayr

**Affiliations:** 1 Department of Pharmacy, Pharmaceutical Biology, Ludwig-Maximilians-University (LMU) Munich, 81377 Munich, Germany; 2 Institute of Pharmacy, Paracelsus Medical University, Strubergasse 21, 5020 Salzburg, Austria; 3 Department of Chemistry, Center for Integrated Protein Science Munich, Technical University Munich, Lichtenbergstraße 4, 85748 Garching, Germany; 4 Institute for Mitochondrial Diseases and Aging at CECAD Research Centre, and Center for Molecular Medicine Cologne (CMMC), Medical Faculty, University of Cologne, Cologne, Germany; 5 Faculty of Medicine, Institute of Anatomy, Ludwig-Maximilians-University (LMU) Munich, 80336 Munich, Germany; 6 Research Unit Apoptosis in Hematopoietic Stem Cells, Helmholtz Zentrum München, 81377 Munich, Germany; 7 Dr. Von Hauner Children’s Hospital, Ludwig Maximilians University (LMU), 80337 Munich, Germany; 8 Institute of Pharmacy, Center for Public Health, Paracelsus Medical University, 5020 Salzburg, Austria; 9 Present Address: Science for Life Laboratory, Department of Oncology-Pathology, Karolinska Institutet, Solna, Box 1031, 171 21 Stockholm, Sweden

**Keywords:** Pharmaceutics, Target validation, Proteomics, Screening, Small molecules, Cancer therapy, Haematological cancer, Cancer therapeutic resistance, Drug development, Targeted therapies

## Abstract

The human mitochondrial ClpXP protease complex (HsClpXP) has recently attracted major attention as a target for novel anti-cancer therapies. Despite its important role in disease progression, the cellular role of HsClpXP is poorly characterized and only few small molecule inhibitors have been reported. Herein, we screened previously established *S. aureus* ClpXP inhibitors against the related human protease complex and identified potent small molecules against human ClpXP. The hit compounds showed anti-cancer activity in a panoply of leukemia, liver and breast cancer cell lines. We found that the bacterial ClpXP inhibitor 334 impairs the electron transport chain (ETC), enhances the production of mitochondrial reactive oxygen species (mtROS) and thereby promotes protein carbonylation, aberrant proteostasis and apoptosis. In addition, 334 induces cell death in re-isolated patient-derived xenograft (PDX) leukemia cells, potentiates the effect of DNA-damaging cytostatics and re-sensitizes resistant cancers to chemotherapy in non-apoptotic doses.

## Introduction

Pharmacological advances have evolved anti-cancer treatment from early, single-agent chemotherapeutics to polychemotherapy and therapies targeting the hallmark features of malignancies, such as multikinase inhibitors and immunological approaches. In acute myeloid leukemia (AML) these progresses have achieved a remission rate upon first-line treatment of 40–80%^1,2^. Nevertheless, acquired drug resistances result in disease relapse and continues to be the principal limiting factor to achieving permanent remission. In fact, until today, standard regimens of AML failed in > 60% of cancer patients due to recurrence^2,3^. Thus, there is an urgent need for innovative targeted drugs that replace or support current therapies and improve the overall survival by preventing, delaying or reverting resistance to anti-cancer agents.

The mitochondrial proteolytic complex ClpXP has recently gained attention in the search for novel therapeutic targets for the treatment of cancer^4^. This prominent proteolytic machinery consists of two stacked heptameric rings of the caseinolytic peptidase ClpP that associate with the hexameric AAA + (ATPase associated with diverse cellular activities) unfoldase ClpX to form a fully functional proteolytic complex^5,6^. In bacteria this complex already represents an attractive drug target due to its central role in virulence regulation and protein homeostasis. While inhibition of ClpP renders harmful human pathogens like *Staphylococcus aureus (S. aureus)* non-virulent, overactivation through selective small molecules induces uncontrolled proteolysis leading to rapid cell death^7–10^. In contrast, the human ClpXP (HsClpXP) analog, which resides in the mitochondrial matrix, remained unexploited as a drug target until recently when an essential link between HsClpP and several leukemic cell lines was established via genetic and chemical knock-downs^4^. There lactone-based inhibitors, originally developed to target *S. aureus* ClpP (SaClpP), could be successfully employed to inhibit the growth of cancer cells in a ClpP-dependent manner. In addition, phenylesters were customized as versatile inhibitors of human ClpP^11^. Most recently it could be shown that the concept of induced cell death by overactivation of ClpP can be translated from bacteria to cancer cells^12,13^. Imipridone ONC201, a highly potent ClpP overactivator, is already in clinical trials for several cancers, further highlighting the enormous potential of ClpP as anticancer target. However, one major challenge is the stable inhibition of the fully assembled ClpXP complex due to conformational control of ClpX over ClpP leading to reduced inhibitor binding^14^. Thus, compounds targeting the whole human ClpXP complex rather than just the ClpP subunits bear a great potential as novel drug candidates. To this end we set out to reinvestigate a previous high-throughput screen (HTS) conducted to identify *S. aureus* ClpXP (SaClpXP) inhibitors, which target human ClpXP^15^. Among several hits, one bacterial ClpXP inhibitor was identified as promising scaffold for the pharmacological inhibition of HsClpXP. Furthermore, the compound exhibited anti-cancer activity, induced cell death in re-isolated patient-derived xenograft (PDX) leukemia cells, potentiated the effect of DNA-damaging cytostatics and re-sensitized chemoresistant cancers to therapy.

## Results

### Chapter 1: Activity of the ClpXP complex is efficiently reduced by 319, 334 and 339

**Figure 1:**
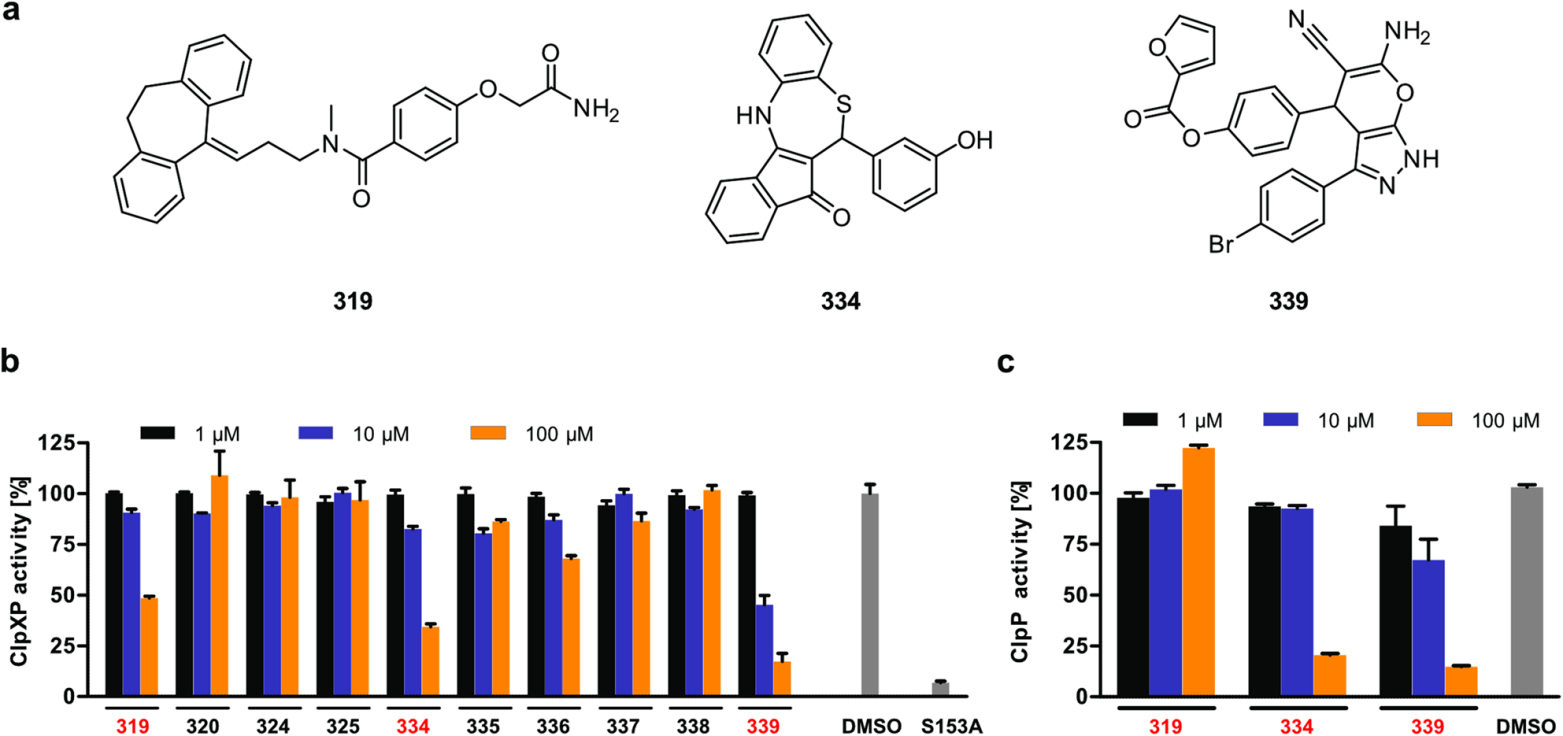
Small molecule 334 is a promising candidate for inhibition of the ClpXP complex. (**a**) The chemical structures of the small molecules 319, 334 and 339 inhibiting the ClpXP complex. For the chemical structure of all molecules tested for inhibition see also Supplementary Fig. S1 online. (**b**) Small molecules with activity towards the bacterial SaClpXP complex were tested for their inhibitory potential against the HsClpXP isoform. The ClpXP enzyme activity was assessed by fluorescence recording of cleaved FITC-casein conjugates (n = 3). (**c**) Promising candidates 319, 334 and 339 were additionally tested for inhibition of the HsClpP peptidase activity. Enzymatic activity of HsClpP was measured by monitoring the cleavage of the fluorogenic substrate Ac-Ala-hArg-2-Aoc-ACC (n = 4).

In a previously conducted HTS, small molecule inhibitors with activity against the ClpXP complex of *S. aureus* were identified (Fig. 1a, Supplementary Fig. S1 online)^15^. This suite of compounds was re-investigated here for the inhibition of the human ClpP peptidase as well as the whole reconstituted mitochondrial ClpXP complex (Fig. 1b,c). Interestingly, compounds 319, 334 and 339 inhibited the protease activity of the fully assembled human complex (Fig. 1b). Corresponding studies on the peptidase level revealed that 334 and 339 impaired ClpP activity while 319 did not show an effect even at the highest concentration (Fig. 1c)^9,11^.

### Chapter 2: ClpXP inhibitor 334 impairs the oncogenic potential of leukemia cells

**Figure 2:**
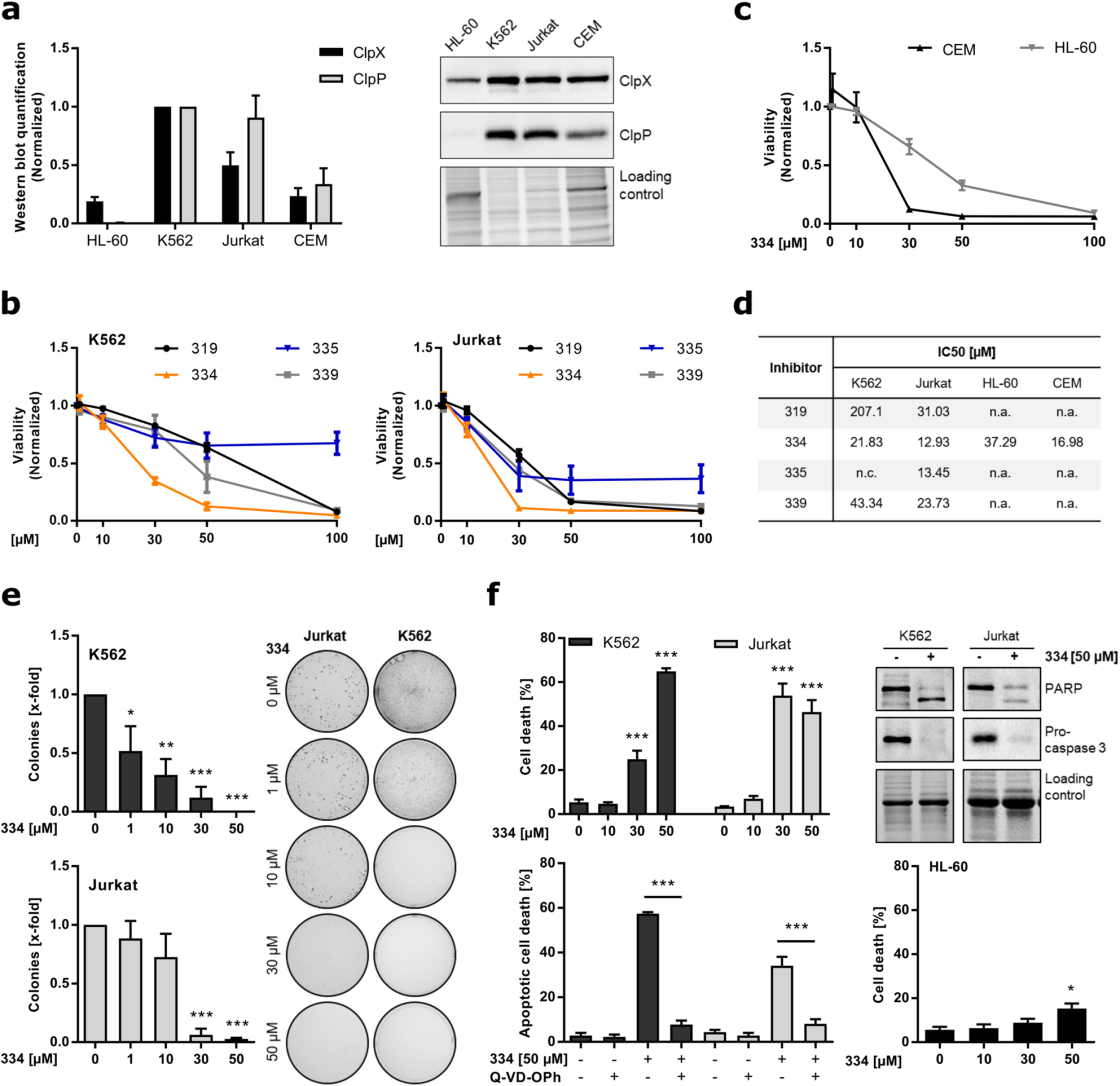
334 treatment impairs viability and colony formation of various leukemia cell lines. (**a**) ClpX and ClpP protein is expressed in various leukemia cell lines. Western blot analysis was performed for ClpX and ClpP in HL-60, K562, Jurkat, and CEM cells. The quantification of ClpX and ClpP protein expression was normalized to K562 and the respective protein load (n = 3). (**b**) 334 has strongest impact on cell viability of K562 and Jurkat cells. Cell viability of K562 and Jurkat cells upon 334 treatment (72 h) was normalized to the DMSO treated control (n = 3). (**c**) 334 treatment decreases cell viability of various leukemia cell lines. Cell viability of HL-60 and CEM cells upon 334 treatment for 72 h was normalized to the DMSO treated control. (**d**) IC_50_ determination of various ClpXP inhibitors. Table shows IC_50_ values calculated by nonlinear regression analysis. Missing data was not assessed (n.a.) or not calculated (n.c.) due to an insufficient inhibitory effect (n = 3). (**e**) 334 impairs clonogenic growth of K562 and Jurkat cells. Colony count of K562 and Jurkat cells normalized to untreated control cells is shown (left panels). Representative images of colonies formed by Jurkat and K562 cells pretreated with 334 for 24 h and grown in the absence of 334 for further 7 days are shown (right panel) (*P < 0.05, **P < 0.01, ***P < 0.001, One-way ANOVA, Tukey’s Multiple Comparison Test, n = 3). (**f**) 334 induces apoptotic cell death in K562 and Jurkat cells. Cell death upon 334 treatment (48 h) was assessed by flow cytometry using Nicoletti assay (upper left panel). Western blot analysis of PARP and procaspase-3 protein expression together with the respective whole protein loading controls are shown for K562 and Jurkat cells treated with 334 (50 µM, 24 h) (n = 3). The apoptotic cell death was assessed for cells treated with 334 (50 µM, 48 h), the pan-caspase inhibitor Q-VD-OPh (20 µM, 30 min) or a combination of Q-VD-OPh pretreatment prior to 334 exposure (50 µM, 48 h) (lower left panel). Cell death of HL-60 cells treated with 334 for 48 h is shown (lower right panel) (*P < 0.05, ***P < 0.001, One-way ANOVA, Tukey’s Multiple Comparison Test, n = 3). For (**a**) and (**f**) full length blots are presented in Supplementary Fig. S8 online. See also Supplementary Figs. S2 and S3 online.

It is known that dependent on the type of malignancy, ClpP overexpression in human cancer correlates with low patient survival^16^. Hence, the abundance of this protease might also dictate the susceptibility of cancer cells to ClpXP-targeting agents. Therefore, the protein expression of the ClpX chaperone and ClpP were assessed in different leukemia cell lines. Both subunits were most prominently expressed in the chronic myeloid leukemia (CML) cell line K562 and in T-cell acute lymphoblastic leukemia (T-ALL) Jurkat cells. ClpP and ClpX occurred to a lower extent in the T-ALL cell line CEM and the lowest abundance was found in the AML cell line HL-60 (Fig. 2a). Thus, the active HsClpXP inhibitors 319, 334, 339 and the SaClpXP inhibitor 335 were subsequently tested for their proliferation inhibiting potential in K562 and Jurkat cells. In both human leukemic cell lines 334 protruded as the most potent inhibitor of viability, whereas 335 appeared to be least effective (Fig. 2b). A reduction of cell viability by 334 was confirmed in HL-60 and CEM cells (Fig. 2c). Notably, a low ClpXP protein expression in HL-60 cells correlated with a low sensitivity towards 334 (IC_50_: 37.29 µM), whereas the viability of Jurkat cells was efficiently reduced (IC_50_: 12.93 µM) (Fig. 2d). The anticancer potential of ClpXP inhibition by 319 and 334 was reinforced in liver (HUH7; HepG2) and breast cancer (MDA; MCF7) cell lines. Thereby, 334 obtained the strongest reduction of viability in the hepatocellular carcinoma (HCC) cell line HUH7 and also impaired cell migration at low concentrations (Supplementary Fig. S2a–c online). In addition, colony formation of K562, Jurkat and HUH7 cells was decreased in a concentration-dependent manner (Fig. 2e; Supplementary Fig. S2d online). However, the susceptibility to ClpXP inhibition may vary among cancer types, as 335 obtained good activity in the tested liver and breast cancer cell lines, whereas in HUH7 cells, 339 exclusively reduced cell migration (Supplementary Fig. S2; Supplementary Fig. S3 online). At high 334 concentrations (50 µM), PARP cleavage and concomitant Caspase-3 activation indicate apoptotic cell death in K562 and Jurkat cells, which was prevented by pretreatment with the pan-caspase inhibitor Q-VD-OPh. However, low ClpXP protein expressing HL-60 cells were refractory to apoptosis induction by 334 (Fig. 2f).

### Chapter 3: 334 induces cell death in patient-derived xenograft (PDX) cells

**Figure 3:**
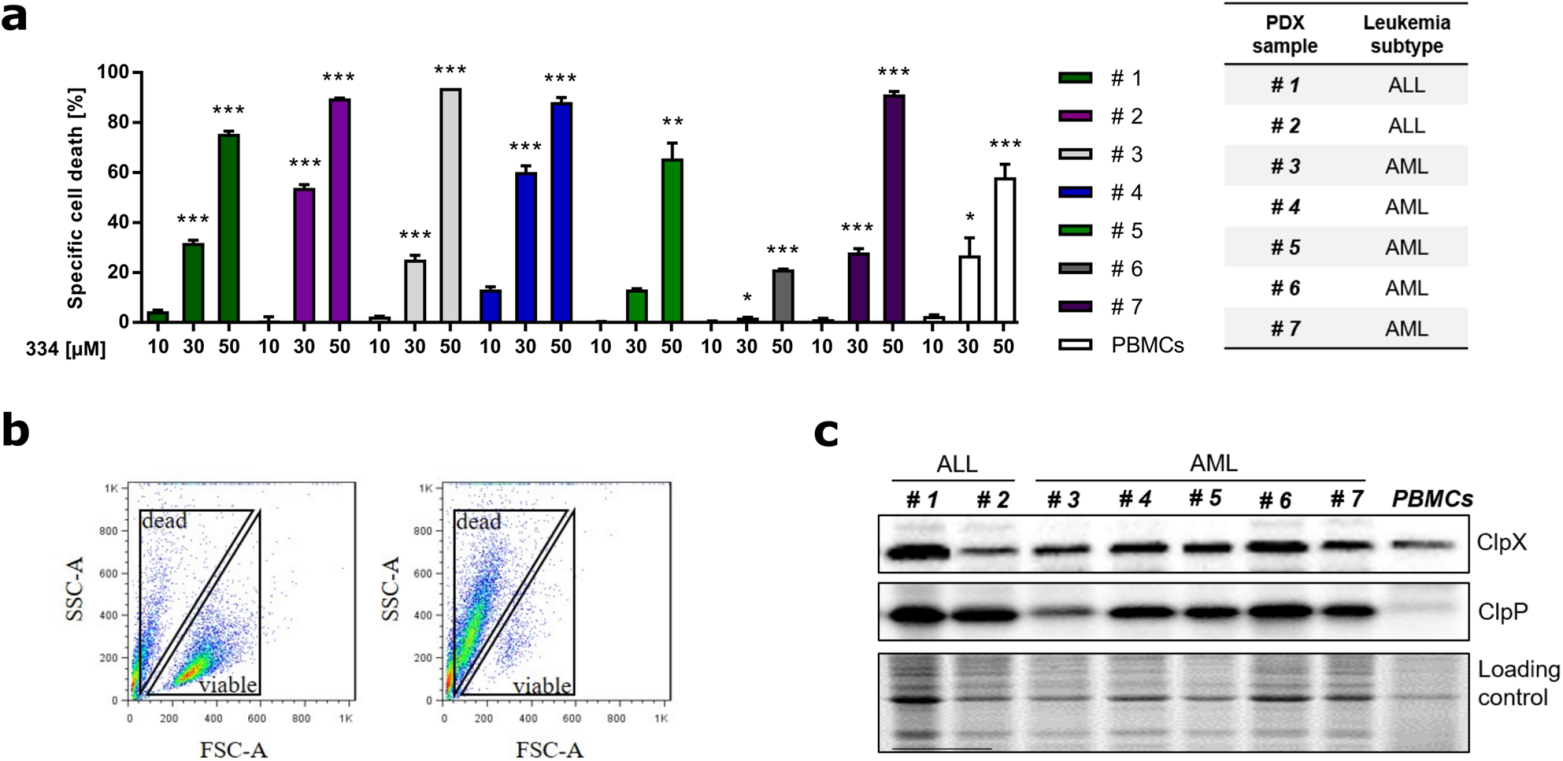
334 induces cell death in ALL and AML PDX cells and PBMCs. (**a**) 334 induces cell death in all tested PDX leukemia cells. Specific cell death of PDX cells (n = 1) and isolated PBMCs (n = 3) treated with 334 for 48 h is shown (*P < 0.05, **P < 0.01, ***P < 0.001, One-way ANOVA, Tukey’s Multiple Comparison Test, n = 1(PDX)/n = 3 (PMBCs), 5 repeated measurements). (**b**) The specific cell death was quantified by flow cytometry as shown by a representative FSC-SSC scatter plot of untreated (left panel) vs. 334 treated (50 µM) PDX cells (right panel). (**c**) ClpX and ClpP protein expression does not correlate with 334 treatment efficiency. Western blot analysis for ClpX and ClpP protein expression in all tested PDX cells and PBMCs is shown (n = 1). Stain-free technology is used as a whole protein loading control. Full length blots are presented in Supplementary Fig. S8 online.

In order to assess the therapeutic relevance, 334 was tested in PDX cells of 5 AML and 2 ALL patients (Supplementary Table S1 online). Therefore, primary tumor cells isolated from the peripheral blood of patients were transduced to establish stable luciferase expression, injected into immune-compromised mice and the development of leukemia was monitored by bioluminescence in vivo imaging^17,18^. AML and ALL PDX cells were re-isolated and harnessed for further ex vivo analysis. Isolated peripheral blood mononuclear cells (PBMCs) were used as healthy control cells. In fact, applying 334 significantly induced cell death in all tested patient-derived cells (Fig. 3a). The specific cell death of PDX cells and PBMCs was determined by flow cytometry via quantification of forward and side scatter plot signals (FSC-SSC). Spontaneous cell death was subtracted from therapy-induced apoptosis as previously described (Fig. 3b)^19^. However, no correlation between ClpXP protein expression with the 334-induced specific cell death was observed in PDX and PBMC cells (Fig. 3c). This suggests that 334 may have additional targets, the heterogeneous genetic background of PDX cells plays a role in the sensitivity to 334 or that its proapoptotic effect is partially dictated by cellular stress conditions.

### Chapter 4: Promotion of cellular stress response and mitochondrial alterations by 334

**Figure 4:**
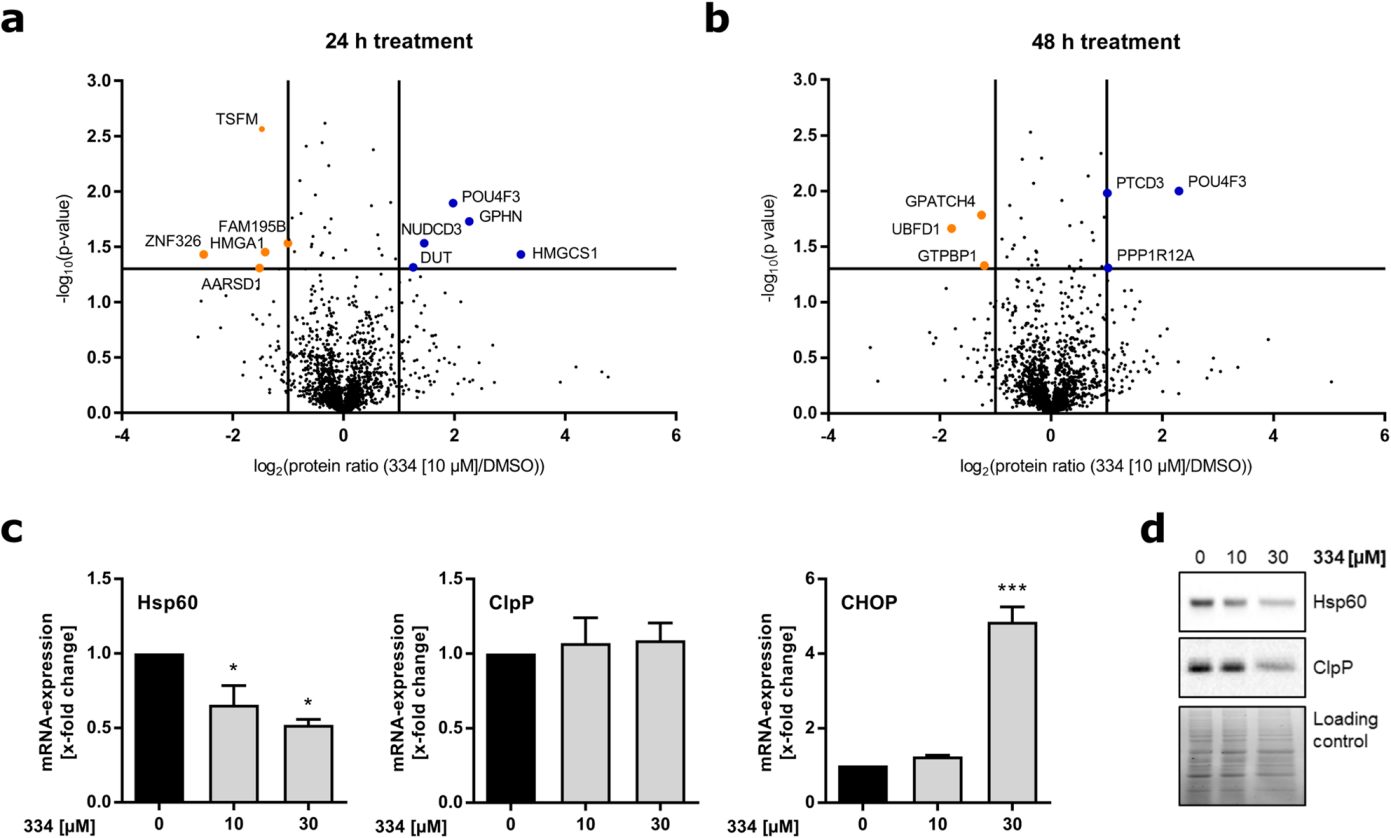
334 induces cellular stress and alters the mitochondrial proteostasis. (**a**) Short-term 334 treatment (24 h) increases unfolded protein binding and decreases DNA repair. (**b**) Further, 334 treatment upon 48 h indicates the induction of the mtUPR. For **(a)** and **(b)** volcano plot analysis of quantitative MS-proteomics by SILAC are shown. Light, medium, and heavy isotope-labeled K562 cells were treated with 334 (10 µM) for the indicated time and compared to DMSO-treated controls (n = 3). Significantly enriched (blue) or depleted (orange) proteins are highlighted. Cut-off criteria: *P* < 0.05; |log_2_(334 [10 µM]/DMSO)|> 1. See also Supplementary Fig. S4a,b, Supplementary Tables S1 and S2 online. (**c**) 334 reduces Hsp60 mRNA expression and induces transcription of CHOP. mRNA levels of Hsp60, ClpP, and CHOP were assessed in 334-treated Jurkat cells after 24 h and normalized to untreated controls. Relative mRNA expression was assessed by normalization to mRNA of the housekeeping gene actin (*P < 0.05, ***P < 0.001, One-way ANOVA, Tukey’s Multiple Comparison Test, n = 3). (**d**) 334 treatment impairs Hsp60 and ClpP protein expression. Western blot analysis shows Hsp60 and ClpP protein levels in Jurkat cells after 334 treatment for 24 h (n = 3). Full length blots are presented in Supplementary Fig. S8 online.

So far, the ClpXP inhibitor 334 has been shown to possess anticancer potential in a panoply of cancer cell lines derived from the hematopoietic system, liver or breast and in leukemic PDX cells. In order to gain further insights into 334’s mechanism of action, we applied quantitative mass spectrometry (MS) using stable isotope labeling with amino acids in cell culture (SILAC). Labeled (light, medium or heavy arginine and lysine) K562 cells were treated with 334 for 24 h or 48 h following quantitative assessment of regulated proteins. Among the significantly altered proteins, mitochondrial stress mediators as well as proteins involved in transcriptional and translational regulation were identified (Fig. 4a,b). In detail, unfolded protein binding (NUDCD3), stress-related proteins (PPP1R12A; GPHN) and enzymes of the mitochondrial lipid and nucleotide metabolism (HMGCS1; DUT) were enriched upon 334 exposure. Notably, the antiapoptotic transcription factor POU4F3, which plays a role in regulation of drug-induced toxicity, was significantly upregulated upon both 24 h and 48 h of 334 treatment^20^. On the other hand, DNA repair (HMGA1), RNA editing or degradation (AARSD1; ZFN326; GTPBP1) and a NF-κB regulator (UBFD1) were found to be depleted (Supplementary Fig. S4a,b; Supplementary Tables S2–S5 online). Upon challenges of persistent mtROS production and potential mitochondrial DNA mutations, mitochondria ensure integrity and functionality by an intrinsic adaptive system, the mitochondrial unfolded protein response (mtUPR)^21^. Thereby, in parallel to NF-κB signaling, the mtUPR initiates transcription of the nuclear-encoded mitochondrial protease ClpP and mitochondrial chaperones, such as the heat shock protein 60 (Hsp60)^22,23^. However, when overactivated, the UPR leads to cell dysfunction and apoptotic cell death via the transcriptional mediator C/EBP Homologous Protein (CHOP)^24^. In fact, via quantitative real-time PCR we revealed a significant increase of CHOP mRNA at high 334 concentrations (30 µM) (Fig. 4c). In addition, both Hsp60 and ClpP protein expression were reduced in Jurkat cells (Fig. 4d), suggesting that ClpXP inhibition could promote an uncompensated mtUPR, which mediates cell dysfunction and apoptosis by CHOP expression.

### Chapter 5: 334 leads to impairment of the mitochondrial respiratory chain

**Figure 5:**
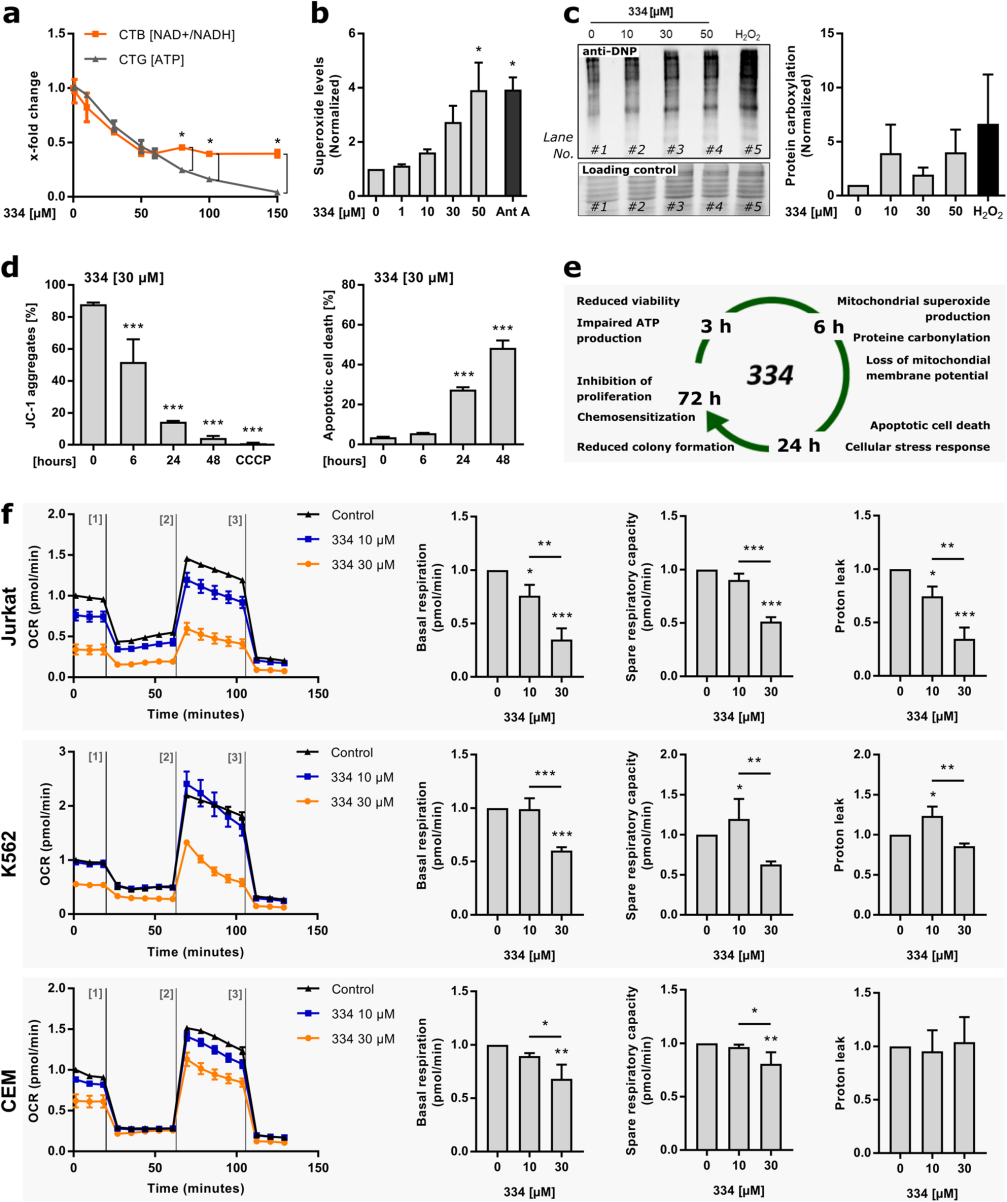
334 promotes cell death by impairing the integrity of the mitochondrial membrane potential (MMP). (**a**) Short-term ClpXP inhibition by 334 reduces the cellular ATP levels. Jurkat cells were treated with 334 (3 h) and the cell viability was assessed by CellTiter-Blue™ (CTB) reagent. At the same time-points cellular ATP levels were measured by CellTiterGlo™ (CTG) reagent and the relative effect was compared to the cell viability at the indicated concentrations (*P < 0.05, t-test, Holm-Sidak method, n = 3). (**b**) 334 treatment drives mitochondrial ROS production. Mitochondrial ROS levels were assessed by flow cytometry using the MitoSOX™ reagent and relative superoxide levels are shown as mean fluorescence intensity normalized to the untreated control. Cells were treated with 334 as indicated (6 h) and the positive control was treated with antimycin A (Ant A) (20 µM, 45 min) prior to measurements (*P < 0.05, One-way ANOVA, Tukey’s Multiple Comparison Test, n = 3). (**c**) 334 promotes protein carbonylation by ROS-mediated oxidation reactions. Western blot shows bands of carbonylated proteins after treatment of Jurkat cells with 334 at the indicated concentrations (6 h). H_2_O_2_ pretreatment was used as a positive control (1 mM, 1 h) (left panel). Protein carboxylation was quantified and normalized to the untreated control (right panel) (One-way ANOVA, Tukey’s Multiple Comparison Test, n = 3). (**d**) Short-term ClpXP inhibition by 334 disrupts the mitochondrial membrane potential without inducing apoptosis. JC-1 dye aggregates indicate mitochondria with intact MMP and were quantified by flow cytometry upon 334 treatment (30 µM) for the indicated time. Carbonyl cyanide 3-chlorophenylhydrazone (CCCP) was used as a positive control for the loss of MMP (left panel). Apoptosis was assessed upon 334 treatment (30 µM) for the indicated time. Percentage of apoptotic cells as assessed by flow cytometry are shown (***P < 0.001, One-way ANOVA, Tukey’s Multiple Comparison Test, n = 3). (**e**) Schematic overview of the impact of 334 treatments on mitochondrial and cellular functions. See also Supplementary Fig. S5 online. (**f**) 334 reduces basal respiration and respiratory capacity of leukemia cells. The oxygen consumption rate (OCR) over time was assessed for Jurkat, K562 and CEM cells untreated or treated with 334 (10 µM and 30 µM) for 24 h via mitochondrial stress test on an Agilent Seahorse XF Analyzer. OCR measurements were performed untreated and after injection of oligomycin (2.5 µM), FCCP (0.5 µM for K562/2.0 µM for Jurkat and CEM) and Rotenone/Antimycin A (0.5 µM). Basal respiration, spare respiratory capacity and proton leak were assessed as described in Supplementary Fig. S6a online. The FCCP titration for all cell lines is shown in Supplementary Fig. S6b online (*P < 0.05, **P < 0.01, ***P < 0.001, One-way ANOVA, Tukey’s Multiple Comparison Test, n = 3).

**Figure 6:**
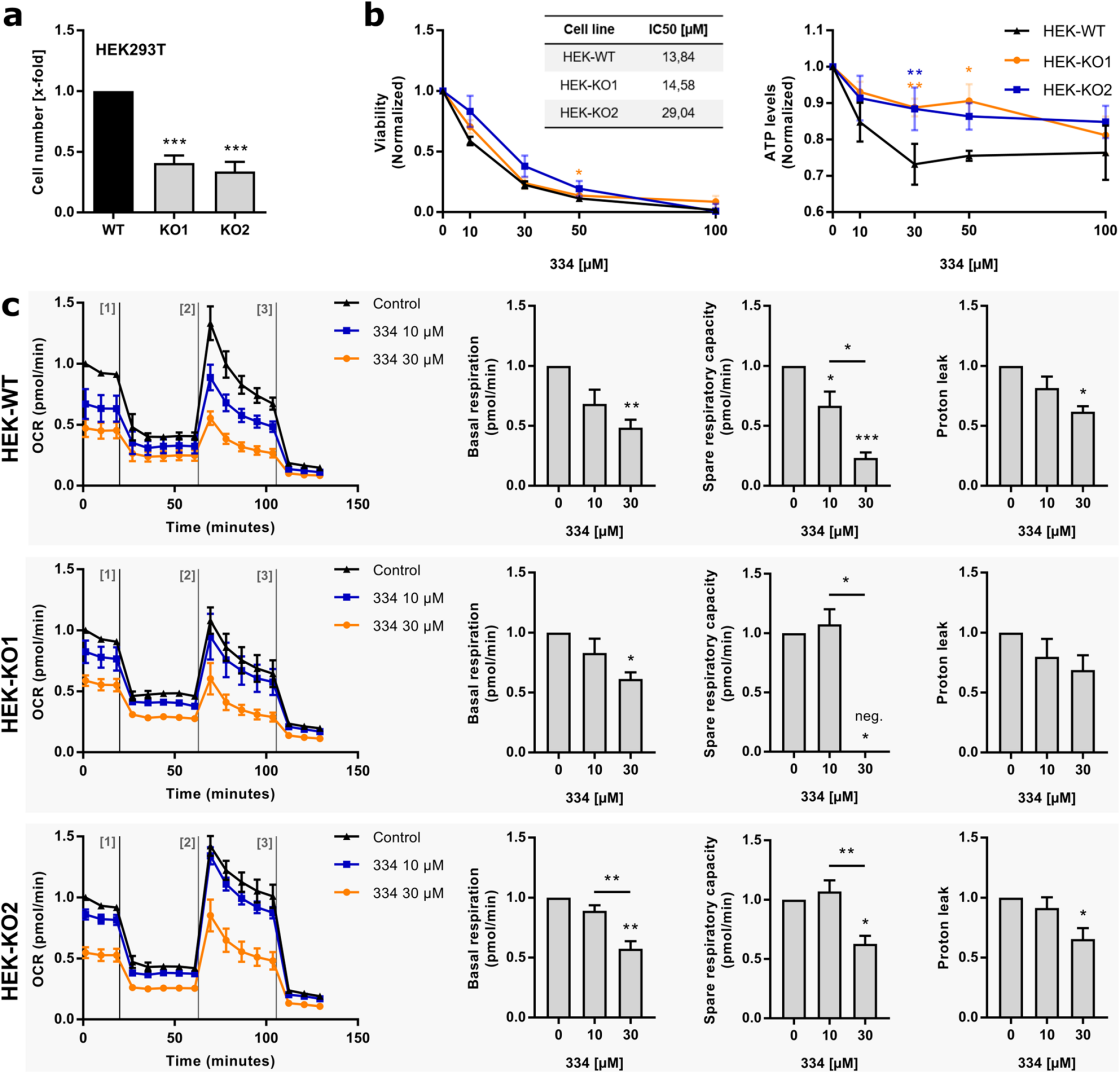
ClpP-KO impairs metabolic impact of 344 treatment. (**a**) Proliferation of HEK293T cells is impaired by knockout (KO) of the ClpXP complex. Cell number of HEK293T cells (HEK-WT) and two distinct ClpP-KO clones^30^ (HEK-KO1, HEK-KO2) was assessed by crystal violet staining 72 h after cell seeding and normalized to HEK-WT (***P < 0.001, One-way ANOVA, Tukey’s Multiple Comparison Test, n = 3). (**b**) An intact ClpXP complex is required for 334-mediated reduction of ATP production. Viability with corresponding IC_50_ values (left panel) and ATP levels (right panel) of HEK-WT, HEK-KO1 and HEK-KO2 cells are shown. Cells were treated with 334 (10 µM, 30 µM, 50 µM, 100 µM) for 3 h (ATP production) or 72 h (Viability) and results were normalized to the untreated control of the respective cell type (*P < 0.05, **P < 0.01, paired t-test comparing HEK-KO1 or HEK-KO2 to HEK-WT, n = 3). (**c**) Respiratory capacity of ClpP-deficient (−/−) HEK (HEK-KO) cells is not altered by low-dose (10 µM) 334 treatment. The OCR over time was assessed for HEK-WT and HEK-KO1/HEK-KO2 cells untreated or treated with 334 (10 µM and 30 µM) for 24 h via mitochondrial stress test on an Agilent Seahorse XF Analyzer. OCR measurements were performed untreated and after injection of Oligomycin (2.5 µM), FCCP (0.5 µM) and Rotenone/Antimycin A (0.5 µM). Basal respiration, spare respiratory capacity and proton leak were assessed as described in (Supplementary Fig. S6a online). The FCCP titration for HEK-WT cells is shown in Supplementary Fig. S6b online (last panel) (*P < 0.05, **P < 0.01, ***P < 0.001, One-way ANOVA, Tukey’s Multiple Comparison Test, n = 3).

The induction of mitochondrial stress in response to 334 treatment suggests further investigations of the mitochondrial function and morphology. The electron transport chain (ETC), which is located within the inner mitochondrial membrane is the major production site of cellular ATP^25,26^. We found that short-term treatment with 334 diminished cellular ATP levels in Jurkat cells with higher efficiency then cell viability, indicating a specific effect of 334 on mitochondrial respiration (Fig. 5a). However, the mitochondrial network obtained no major alterations in cellular localization and the overall mitochondrial integrity remained intact upon ClpXP inhibition (Supplementary Fig. S5a,b online). At physiological conditions, the ETC produces low concentrations of reactive oxygen species (ROS) as a byproduct of mitochondrial respiration^27^. However, ETC deficiency leads to excessive mtROS production, which affects mitochondrial functionality and cell fate^28,29^. It has recently been shown that in order to avoid oxidative stress, ClpXP specifically removes damaged subunits of the ETC complex I^30^. In fact, mitochondrial ROS production was increased by 334 in a concentration-dependent manner (Fig. 5b). Further, high superoxide levels promoted oxidation reactions and protein carbonylation (Fig. 5c). Protein carbonylations are covalent modifications with carbonyl residues that occur upon oxidative stress and target proteins for selective degradation by the 26S proteasome^31,32^. In general, elevated protein carbonylation is accompanied by diminished ETC activity and a reduction of the mitochondrial membrane potential (MMP) without affecting mitochondrial number, area, or density^33^. In line, a loss of the MMP, which occurs as an initial event of intrinsic cell death, was observed already after 6 h of ClpXP inhibition and led to high levels of apoptosis at later time points (Fig. 5d)^25,34^. In summary, low ATP levels and high mtROS production indicate impairment of the ETC in an early phase of inhibitor treatment. A disturbed MMP, enhanced protein carbonylation, protein aggregation with activation of the mtUPR and the induction of intrinsic apoptosis are the consequence (Fig. 5e)^35,36^. In order to pinpoint the metabolic impact of 334 on reduced ETC activity, a mitochondrial stress test was performed in a panoply of leukemia cell lines. In fact, we found that 334 diminished basal respiration and the respiratory capacity of Jurkat, K562 and CEM cells (Fig. 5f, Supplementary Fig. S6 online). Next, we investigated if the ClpXP complex is the target of 334, which confers the inhibitory effect on the mitochondrial metabolism. Therefore, ClpP-deficient HEK293T clones (HEK-KO1 and HEK-KO2), which have been previously characterized, were compared to HEK-WT cells^30^. We found that ClpP-deficient cells are less sensitive towards a reduction of ATP levels by short-term 334 treatment (3 h). However, a reduction of proliferation (72 h) might be partially caused by off-target effects (Fig. 6a,b). Further, the reduction of the spare respiratory capacity was shown to be partially mediated by targeting the ClpXP complex, as upon low-dose 334 treatment no significant reduction was observed in HEK-KO cells in contrast to HUH7-WT cells (Fig. 6c). In summary, even though, off-target effects cannot be excluded, we found a direct involvement of ClpXP in mediating the metabolic alterations of 334.

### Chapter 6: Chemo-sensitivity of leukemia cells is enhanced by 334 treatment

**Figure 7:**
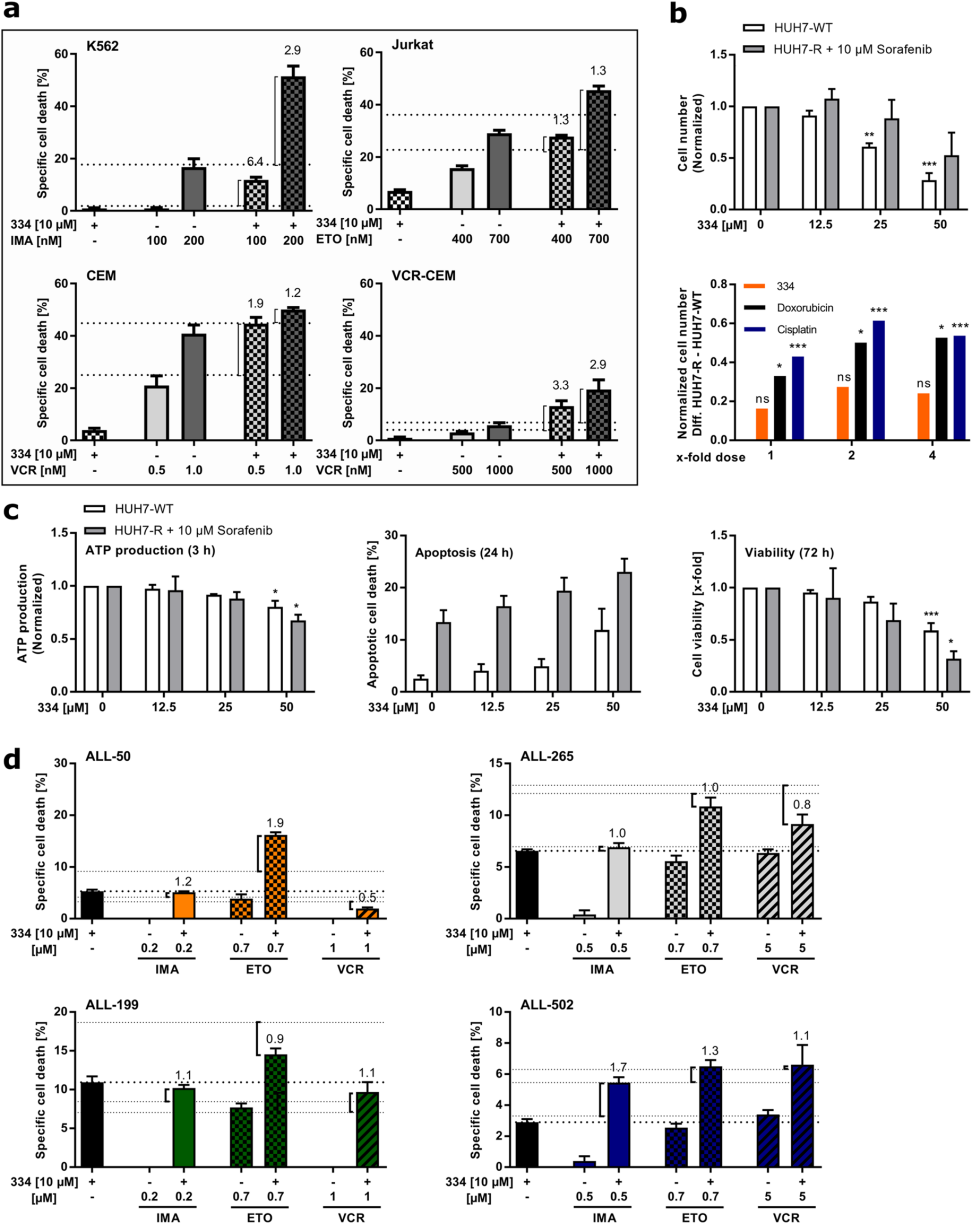
Cancer cell sensitization towards chemotherapy. (**a**) 334 sensitizes leukemia cells towards chemotherapy. Specific cell death induced in K562, Jurkat, CEM, and vincristine-resistant CEM cells (VCR-CEM) is shown. Cells were treated as indicated with 334 (10 µM), imatinib (IMA; K562) etoposide (ETO; Jurkat), and vincristine (VCR; CEM and VCR-CEM) alone or in combination for 48 h. Cell death was assessed by flow cytometry using Nicoletti assay. Bliss values (BVs) were calculated for combined cytostatic therapies and synergism is illustrated by response additivity (dashed lines). BVs > 1.05 or a combinational drug effect above the dashed lines indicate synergism (n = 3). (**b**) 334 re-sensitizes sorafenib-resistant HUH7 cells (HUH7-R) cells, which obtained broad chemotherapeutic cross-resistance. HUH7-R were generated from wild-type HUH7 cells (HUH7-WT) by drug exposure and maintained in 10 µM sorafenib. Proliferation rates within 72 h of 334 treatment were assessed by crystal violet staining and normalized to the untreated control (upper panel). Proliferation rates of cytostatic treatment were previously shown^40^. The difference in growth rates obtained by HUH-R and HUH-WT cells was calculated and compared among 334, cisplatin and doxorubicin treatments (lower panel, see also Supplementary Fig. S7c online) (*P < 0.05, ***P < 0.001, One-way ANOVA, Tukey’s Multiple Comparison Test comparing concentrations, t-test comparing cell types, n = 3). (**c**) 334 overcomes broad chemotherapeutic cross-resistance of sorafenib-resistant HUH7-R cells by impairing the mitochondrial integrity. ATP-production (3 h), apoptosis (24 h) and cell viability (72 h) were assessed upon 334 treatment at the indicated time points (*P < 0.05, ***P < 0.001, One-way ANOVA, Tukey’s Multiple Comparison Test comparing concentrations, n = 3). (**d**) Low-dose 334 treatment sensitizes PDX leukemia cells towards chemotherapy. Specific cell death of PDX cells treated with 334 (10 µM), etoposide (700 nM)/imatinib (500 nM)/vincristine (5 nM) or a combination of both for 48 h is shown. The specific cell death was quantified by flow cytometry via FSC-SSC scatter plot analysis. BVs were calculated for combined cytostatic therapies and synergism is illustrated by response additivity (dashed lines). BVs > 1.05 or a combinational drug effect above the dashed lines indicate synergism. (*P < 0.05, One-way ANOVA, Tukey’s Multiple Comparison Test, n = 1, duplicates).

Low-doses of 334 triggered mitochondrial stress in K562, Jurkat and CEM leukemia cells and may thus be harnessed to increase the cancer cells’ susceptibility to apoptosis and to sensitize them towards chemotherapeutic treatment^37,38^. In fact, K562 and Jurkat cells were synergistically sensitized towards the tyrosine kinase inhibitor imatinib and the topoisomerase II inhibitor etoposide when applied in combination therapy with non-cytotoxic doses of 334 (10 µM). Further, 334 promoted synergistic cell death in CEM cells treated with vincristine and even re-sensitized vincristine-resistant (VCR)-CEM cells towards therapy (Fig. 7a)^39^. Of note, chemo-sensitivity towards etoposide or cytarabine was not enhanced in HL-60 cells, which we showed to be refractory towards 334 in viability and cell death assays (Supplementary Fig. S7a online). To expand the clinical relevance to the chemotherapeutic re-sensitization of other malignancies, 334 was tested in a previously established sorafenib resistance HCC cell model^40^. The sorafenib-resistant HUH7-R HCC cells were refractory to 10 µM sorafenib and obtained broad chemotherapeutic cross-resistance. Further, HUH7-R cells have been shown to possess ER stress with enhanced proteasomal degradation of mitochondrial proteins and are therefore especially vulnerable to the inhibition of proteostasis which is targeted by the ClpXP inhibitor 334^40^. Interestingly, a combination of sorafenib with 334 achieved cytotoxicity in HUH7-R cells comparable to the parental, non-resistant HUH7-WT cell line and obtained a stronger growth reduction than a combination with doxorubicin, cisplatin, vincristine or gefitinib (Fig. 7b; Supplementary Fig. S7c online). The chemo-sensitizing effect of 334 is thereby comparable to a combination treatment with the Lon-protease inhibitor bardoxolone methyl (CDDO-ME), which is known to target the mitochondrial Lon protease and affect mitochondrial proteostasis (Supplementary Fig. S7c online)^41^. Further, in this sorafenib resistance cell model we confirmed the reduction of ATP production in an early phase after treatment with subsequent induction of apoptosis and reduction of cell viability, thus substantiating the anticancer potential of 334 via inhibition of the ETC (Fig. 7c). In order to substantiate the clinical impact of this chemo-sensitizing potential, PDX leukemia cells were treated with 334, a chemotherapeutic compound or a combination of both for 48 h. In fact, low-dose 334 treatment (10 µM) partially sensitized PDX cells to imatinib, etoposide and vincristine treatment (Fig. 7d).

## Discussion

Cellular and mitochondrial proteostasis were recently reported as a new target for cancer treatment. The proteasome inhibitor bortezomib (VELCADE) is clinically approved for patients with multiple myeloma as first-line treatment and second-line therapy for relapsed or refractory disease and in cytostatic combination regimens^42,43^. In addition, mitochondrial chaperones increasingly drew attention as the Hsp90-binding small molecule gamitrinib was shown to impair stability of the ETC and regulate mitochondrial cell death^44,45^. Hsp60, on the other hand, supports folding and assembly of protein precursors in the mitochondrial matrix and restores proteostasis in the course of the mtUPR^21,46^. Interestingly, in a large number of tumors, Hsp60 crosstalks with the mitochondrial apoptosis machinery and inhibits tumor suppressors^47^. Hence, mitochondrial chaperons evoked as promising target structures to counteract the development and progression of malignancies. Herein, we focus on another crucial part of the mitochondrial proteostasis maintaining network, the protease ClpXP, as a novel and druggable target for cancer therapy.

Recently, involvement of ClpXP was demonstrated in tumorigenesis and progression of AML, prostate, and breast cancer^4,48^. However, thus far predominantly electrophilic inhibitors such as beta-lactone and phenylester have been reported^4,11^. In general, these ClpP subunit inhibitors suffer from limited stability and therefore weaker effects on tumor proliferation and clonogenic growth was observed in comparison with previous gene silencing experiments^11,48^. We hypothesized that ClpXP inhibition by compounds with enhanced plasma stability could be a superior strategy. Repurposing of bacterial ClpXP inhibitors revealed 334 to block the reconstituted mitochondrial protease with anti-proliferative effects on cancer cell-lines. Although the engagement with other cellular targets cannot be excluded, we observed a correlation between low ClpXP protein expression in HL-60 and corresponding poor sensitivity towards the ClpXP inhibitor 334, suggesting a selective effect on cells expressing the target protein. However, PDX and HUH7-R cells revealed no obvious correlation between ClpXP levels and 334 sensitivity, which may additionally be attributed to further off-targets as well as cellular stress conditions paired with an insufficient antioxidant defense^40^. Moreover, ClpP /- KO cells showed impaired proliferation and were less sensitive to the reduction of ATP production by 334 treatment compared to the parental HEK-WT cell line, emphasizing that 334 directly targets the ClpXP complex and thereby impairs the mitochondrial metabolism. Nonetheless, the drug-mediated reduction of the basal respiration was comparable for HEK-WT and HEK-KO cells, indicating off-target cytotoxicity of 334.

Notably, PBMCs possess comparable ClpXP expression and 334 response as their leukemic PDX counterparts. We conclude that the use of 334 in cell death-inducing concentrations involves a high risk of adverse events and requires local or tumor-targeted drug application. However, in non-apoptotic doses, 334 treatment efficiently sensitizes leukemic cells towards imatinib, etoposide, or vincristine and reestablishes the sorafenib responsiveness of HUH7-R cells^49^. In summary, this promising chemo-sensitizing potential of 334 may be harnessed in combination therapies, in order to lower the drug dose and to minimize related side-effects.

In general, the transcription of chaperones and proteases, such as Hsp60 and ClpP, is induced by the mtUPR, which promotes cell survival by preventing aberrant proteostasis and organelle damage^21,50^. However, upon 334 treatment, leukemic cells exhibit low Hsp60 levels and ClpP expression is not increased, indicating insufficient prosurvival mtUPR signaling^16^. In addition, proteomics indicates that 334 promotes a cellular stress response, while attenuating DNA repair and RNA turnover^51^. Interestingly, the transcriptional regulator POU4F3, which potentially counteracts chemotherapy-induced apoptosis, was found to be upregulated upon both 24 h and 48 h of 334 treatment and may partially compensate the mtUPR^20^. Hence, a combined use of ClpXP inhibitors with DNA-damaging agents may optimize the obtained anti-tumor effects. However, optimal drug partners for 334 remain to be identified.

To date, the small molecule 334 faces major obstacles for clinical translation. First, the identified ClpXP inhibitors possess low cancer cell selectivity and potential off-target effects, which limit the application as monotherapy in cytotoxic doses and requires further structural optimization and target validation. Second, there is no clinical experience on the toxicity profile and potential side-effects of ClpXP inhibition in vivo. We hypothesize that a high mitochondrial mass in healthy tissues, such as liver, heart, and muscles, may correlate with a high susceptibility to ClpXP inhibitors. On the other hand, identifying malignancies which, according to their metabolic profile, likely respond to ClpXP inhibition, could be of particular interest for personalized cancer therapy. In fact, we found that HCC cells were more sensitive towards ClpXP inhibitors compared to leukemia cells and therefore suggest that especially tumors with a strong dependency on oxidative phosphorylation are druggable with high efficiency. This may further be harnessed for the eradication of stem-cell like and quiescent cancer cells, which often account for tumor recurrence, aggressiveness and therapy resistance^52–54^.

In summary, we identified novel human ClpXP inhibitors as potential drug scaffolds and present 334 as an efficient combinational agent to common cytostatics, in order to enhance their anti-cancer potential and to regain the sensitivity of chemo-resistant cancers.

## Materials and methods

### Cell lines

#### Cell authentication

The CML cell line K562 was purchased from the Leibniz Institute DSMZ (Braunschweig, Germany). T-ALL Jurkat cells were kindly provided by P. H. Krammer and H. Walczak (Heidelberg, Germany). Vincristine-resistant CEM/VCR-R cells were previously characterized^39^ and both CCRFCEM (CEM) and CEM/VCR-R cells were kindly provided by Prof. Maria Kavallaris (Sydney, Australia). HL-60, MCF-7 and HepG2 cells were purchased from ATCC (Manassas, VA, USA). HUH7 HCC cells (JCRB0403) were obtained from the Japanese Collection of Research Bioresources (Osaka, Japan) and the human mammary carcinoma cell line MDA-MB-231 (MDA) was purchased from Cell Lines Service (Eppelheim, Germany). The sorafenib-resistant HUH7-R cell line was generated by continuous exposure of HUH7 cells to increasing concentrations of sorafenib (BAY 43-9006, Enzo Life Sciences GmbH, Lörrach, Germany) as previously described^40^.

#### Culture conditions

For the cultivation of HUH7-WT, HUH7-R, HepG2, MCF7 and MDA cells, DMEM medium (PAN Biotech GmbH, Aidenbach, Germany) was used. HUH7-R cells were additionally cultured in the presence of 10 µM sorafenib to maintain resistance. HL-60 cells were cultured in IMEM medium (PAN Biotech GmbH, Aidenbach, Germany). K562, CEM and CEM/VCR-R cells were cultured in RPMI 1640 (PAN Biotech GmbH, Aidenbach, Germany) and the medium of Jurkat cells was additionally supplemented with pyruvate (100 mM). All media were supplemented with 10% FCS (PAA Laboratories GmbH, Pasching, Austria) and cells were cultured at 37 °C with 5% CO_2_ in constant humidity. Before cell seeding, culture flasks, multiwell plates and dishes of adherent cell lines were coated with collagen G (0.001% in PBS, Biochrom AG, Berlin, Germany).

### Human ClpP and ClpXP assays

Human ClpX and ClpP were expressed and purified as described previously ^64^. Human ClpXP activity measurements were carried out in a total reaction volume of 100 µL in black, flat-bottom 96-well plates. All data were recorded in triplicates. First 1 µL of 100 × compound stock in DMSO or 1 µL DMSO as a control was added to the wells (fc_DMSO_ = 1% (v/v)). Per well 25 µL 4 × reaction buffer (100 mM Hepes pH 7.6, 30 mM MgCl_2_, 800 mM KCl, 4 mM DTT, 40% (v/v) glycerol), 10 µL 10 × ATP regeneration mix (100 mM Hepes pH 7.0, 40 mM ATP, 160 mM creatine phosphate, 200 U/mL creatine kinase) and 3.0 µM ClpP and 2.8 µM ClpX (monomeric concentrations) were added. The reaction volume was finally adjusted with ddH2O to a volume of 80 µL. The reaction mixtures were incubated in a temperature equilibrated TECAN infinite M200 pro at 37 °C for 30 min. Subsequently, the reaction was started by addition of 20 µL substrate mixture (5:2:1 mix casein (2 mg/mL in PBS)/FITC-casein (1 mg/mL in PBS)/PBS, pH 7.4). The fluorescence signal of the cleaved FITC was recorded over 140 min (excitation: 494 nm, emission: 525 nm, gain 65). The slope of the signal was calculated for t = 800 − 2450 s by linear regression using Microsoft Excel. DMSO-treated control samples were set to 100% activity and all other samples normalized to this value.

The residual hClpP peptidase activity was measured upon treatment with inhibitors by monitoring the cleavage of the fluorogenic substrate Ac-Ala-hArg-(*S*)-2-aminooctanoic acid-7-amino-4-carbamoylmethylcoumarin (Ac-Ala-hArg-2-Aoc-ACC, custom-synthesis by *Bachem*) as described previously^54^. In a black 96-well plate (*Greiner*) 1 μL inhibitor or DMSO as control was aliquoted in four replicates in three different concentrations (100 × stocks in DMSO, final concentrations in assay: 1 μM, 10 μM, 100 μM). Subsequently, 98 μL enzyme buffer mix (final concentration of hClpP 0.4 μM in assay buffer 50 mM HEPES, 300 mM KCl, 1 mM DTT, 15% (v/v) glycerol, pH = 7.5) was added to the wells and incubated at 37 °C for 15 min. Kinetic measurement (1 min intervals) was started after adding 1 μL of peptide substrate Ac-Ala-hArg-2-Aoc-ACC (20 mM stock in DMSO, final concentration 200 μM). Fluorescence of the cleaved dye was detected for 60 min at 37 °C using a *Tecan Infinite M200 pro* (excitation: 380 nm; emission: 430 nm, manual gain: 100). The slope of the fluorescence over time signal was calculated in the time interval t = 420 − 1500 s via linear regression using GraphPad Prism. The residual activity of inhibitor treated protein was determined in comparison to DMSO treated control samples which were normalized to 100% activity.

### Isolation of PBMCs and cultivation of PDX cells

#### Isolation of PBMCs

Peripheral blood mononuclear cells (PBMCs) were isolated from anticoagulated blood (EDTA) provided by Prof. Johanna Pachmayr (Institute of Pharmacy, Paracelsus Medical University). Ficoll-Paque PLUS medium (GE Healthcare Life Sciences, Freiburg, Germany) was used for PMBC isolation from peripheral blood by gradient centrifugation according to the manufacturer’s protocol. Isolated PBMCs were cultured in RPMI 1640 medium supplemented with 20% FCS.

#### In vivo amplification of patient-derived xenograft (PDX) cells

PDX acute lymphoid (ALL) and acute myeloid leukemia (AML) were generated and amplified as previously described^17,55^. In short, patient-derived cells were serially transplanted into the tail vein of 6–12 week old male or female *NOD scid gamma mice* (NSG; The Jackson Laboratory, Bar Harbour, ME, USA). At late stage leukemia, PDX cells were re-isolated from bone marrow and spleen, washed in PBS, and used for ex vivo studies using respective growth media 56.

#### Approval of animal experiments

All animal trials were performed in compliance with the ARRIVE guidelines (https://arriveguidelines.org) and in accordance with the current ethical standards of the official committee on animal experimentation. Written approval was obtained by the government of Upper Bavaria in Germany (ROB-55.2Vet-2532.Vet_02-16-7). Mice were maintained under specific pathogen-free conditions in the research animal facility of the Helmholtz Zentrum München, Munich, Germany. Animals had free access to food and water, and were housed with a 12-h light–dark cycle and constant temperature.

### Quantitative full proteome analysis using stable isotope labeling with amino acids in cell culture (SILAC)

Cells were grown in a humidified atmosphere at 37 °C and 5% CO_2_. Cells were passaged 6 times in appropriate SILAC-medium (PAA) supplemented with 10% dialyzed FCS, 2 mM l-glutamine (PAA), 50 mg [^13^C_6_, ^15^N_4_] l-argenine × HCl, and 50 mg [^13^C_6_, ^15^N_2_] l-lysine × HCl resulting in “heavy” cells (10/8; 48 h treatment) or [^13^C_6_] l-argenine × HCl, and [4,4,5,5-D_4_] l-lysine × HCl (CIL) resulting in “medium” cells (6/4; 24 h treatment) or l-argenine × HCl, and l-lysine × HCl (CIL) resulting in “light” cells (0/0; DMSO control). Cells were plated out on 15 cm dishes and grown to 90% confluency. Cells were treated with 10 µM compound 334 or DMSO as control for 24 h or 48 h. All experiments were carried out in triplicates. After incubation the cells were washed with PBS and scraped off the culture dishes and pelletized at 600 g for 5 min at 4 °C. The cells were then resuspended in PBS with 1% (v/v) NP40 and 1% (w/v) sodium deoxycholate and incubated for lysis for 15 min on ice. The insoluble fraction was pelletized (13,000 rpm, 20 min, 4 °C) and the supernatant was retained for further analysis. Protein amounts were quantified using BCA (bicinchoninic acid) assay according to the manufacturer’s instructions. Heavy, medium and light samples for each replicate were combined at equal protein amounts. All reagents from this point on were mass spectrometry grade. Proteins were precipitated by addition of fourfold excess aceton (− 80 °C) and incubation at − 20 °C overnight. Precipitated proteins were sedimented for 15 min at 16,900 g. Pellets were washed twice with 1 mL MeOH (− 80 °C) and resuspended in 500 µL digestion buffer (7 M urea, 2 M thiourea in 20 mM HEPES) by sonication (10 s at low intensity). The samples were reduced with 0.4 µL 0.5 M DTT (45 min at 37 °C and 450 rpm), alkylated with 2 µL 550 mM iodoacetamide (30 min at RT and 450 rpm in the dark) and the alkylation was quenched by addition of 1.6 µL 0.5 M DTT (30 min at RT at 450 rpm). Pre-digestion was conducted by incubation with 1:300 (w/w) Lysyl Endopeptidase C (0.5 µg/µL; Wako) for 4 h at 37 °C and 450 rpm. 600 µL 50 mM TEAB (Triethylammonium bicarbonate buffer) were added and the samples were digested with 1:300 (w/w) µL trypsin (0.5 µg/µL; Sequencing Grade, Promega) overnight at 37 °C and 450 rpm. Digestion was stopped and pH adjusted to < 3 by addition of formic acid to 1% (v/v) final. The samples were desalted on 50 mg SepPak cartridges (Waters) using a vacuum manifold. Cartridges were activated with 1 mL ACN and washed with 1 mL elution buffer (80% (v/v) ACN; 0.5% (v/v) formic acid) and thrice with 1 mL 0.5% (v/v) formic acid. Samples were loaded slowly by gravity flow and the cartridges were washed five times with 1 mL 0.5% (v/v) formic acid. Peptides were eluted from the cartridges with two times 250 µL elution buffer. Peptides were evaporated *in vacuo* and re-dissolved in water containing 1% formic acid previous to LC–MS/MS analysis. Prior to measurement the peptides were resuspended in 1% (v/v) formic acid to achieve a concentration of 4 µg/µL peptide sample (calculated according to the BCA assay) and filtered through a 0.22 µm PVDF filter. Samples were analyzed via LC–MS/MS using a UltiMate 3000 nano HPLC system (Thermo Fisher Scientific) equipped with an Acclaim C18 PepMap100 75 μm ID × 2 cm trap (Thermo Fisher Scientific, Cat# ES803A) and an Acclaim PepMap RSLC C18 separation column (75 μm ID × 50 cm, Thermo Fisher Scientific, Cat# 164535) coupled to an EASY-source (spray voltage 1600 V, sweep gas off) equipped Thermo Fisher LTQ Orbitrap Fusion mass spectrometer (Thermo Fisher Scientific). Samples were loaded onto the trap column at a flow rate of 5 μL/min with aqueous 0.1% trifluoracetic acid (TFA) and then transferred onto the separation column at 0.3 μL/min. Buffers for the nano-chromatography pump were aqueous 0.1% FA (buffer A) and 0.1% FA in acetonitrile (ACN, buffer B). Samples were separated using a gradient raising buffer B from 5 to 22% in 112 min, followed by a buffer B increase to 32% within 10 min. Buffer B content was further raised to 90% within the next 10 min and held another 10 min at 90%. Subsequently buffer B was decreased to 5% and held until end of the run (total: 152 min). During sample separation ion transfer tube temperature was et to 275 °C and MS full scans were performed at 120,000 resolution in the orbitrap with quadrupole isolation. The MS instrument was operated in a 3 s top speed data dependent mode. The scan range was set from 300 to 1500 m/z with 60% RF lens amplitude. The automatic gain control (AGC) target was set to 200,000, the maximum ion injection time was 50 ms and internal calibration was performed using the lock mass option. Peptides with intensity higher than 5000 and charge state 2–7 were fragmented with higher-energy collisional dissociation (HCD) (30%). Dynamic exclusion time was set to 10 ppm low and high mass tolerance. MS2 scans were recorded in the ion trap operating in rapid mode. The isolation window was set to 1.6 m/z and the AGC target to 1.0e4 with maximum injection time of 35 ms. Ions were injected for all available parallelizable time. Data were analyzed using MaxQuant software using standard settings for SILAC quantification experiments^56^. Searches were performed against a UniProt database of Homo sapiens proteome (taxon identifier: 9606). Data were further processed using Perseus software^57^. Proteins only identified by site, in reverse database search or marked as potential contaminants were excluded from further analysis. Protein ratios were log_2_-transformed, the replicates were grouped and only proteins with valid values in at least 2 out of the 3 replicates were included in further analysis. Data were analyzed by two-sided one sample Student’s t-test with Benjamini–Hochberg correction (false-discovery rate = 0.05). t-test significance was plotted over log_2_-fold protein ratio using GraphPad Prism.

### Immunoblotting

Proteins were separated via SDS-PAGE^58^, transferred to a PVDF membrane (Immun-Blot, Bio-Rad, Munich, Germany) and incubated with one of the following primary antibody diluted in 5% BSA in PBS overnight at 4 °C: β-actin (mouse, 1:1000/Cat. #MAB1501, Merck, Darmstadt, Germany), Caspase-3 (rabbit, 1:1000/Cat. #sc-7148, Santa Cruz Biotechnology, Dallas, TX, USA), ClpP (mouse, 1:1000/Cat. #ab56455, Abcam, Cambridge, UK), ClpX (rabbit, 1:500/Cat. #ab123110, Abcam, Cambridge, UK), Hsp60 (goat, 1:1000/Cat. #sc-1052, Santa Cruz Biotechnology, Dallas, TX, USA) or PARP (rabbit, 1:1000/Cat. #9542, Cell Signaling Technology, Danvers, MA, US). Proteins were visualized using horseradish peroxidase (HRP) coupled secondary antibodies and ECL solution containing 2.5 mM luminol. The following secondary antibodies diluted in 5% BSA in PBS were incubated with the membrane for 2 h at RT: Goat-anti-rabbit IgG(H + L)-HRP conjugate (1:1000/Cat. #111-035-144, DIANOVA GmbH, Hamburg, Germany), goat-anti-mouse IgG1-HRP conjugate (1:1000/Cat. #sc-2005, Santa Cruz Biotechnology, Dallas, TX, USA) and donkey-anti-goat IgG-HRP conjugate (1:1000/Cat. #ab97120, Abcam, Cambridge, UK). Chemiluminescence was detected with the Chemidoc Touch Imaging system (Bio-Rad, Munich, Germany) and the protein expression was quantified using Stain-free technology and the Image Lab Software. This technique enables a quantification of the whole lane protein, and therefore can be used for the normalization of protein bands^59^. The protein expression was normalized to the protein load or the respective β-actin protein expression.

### Cell viability assays

Cell viability was measured using CellTiter-Blue and CellTiter-Glo Luminescent Cell Viability Assay (Promega, Madison, WI, USA) according to the manufacturer’s protocol. Therefore, cells were seeded in 96-well plates and treated with DMSO or respective compounds in the indicated concentrations for the indicated time. Cellular ATP levels were measured via luminescence signal after the addition of CellTiter-Glo using an Orion II microplate luminometer (Titertek Berthold, Pforzheim, Germany). For the determination of cell viability CellTiter-Blue reagent was added to the cells and after four hours the fluorescence intensity was measured using SpectraFluor PlusTM (Tecan, Crailsheim, Germany).

### Colony formation assay

Cells were pretreated with DMSO or compounds in the indicated concentrations for 24 h before being reseeded in 12-well plates (5 × 10^3^ cells/ml) in colony formation medium (growth medium + 40% FCS + 0.52% methylcellulose). After a 7-day incubation MTT (3-(4,5-dimethylthiazol-2-yl)-2,5-diphenyltetrazoliumbromide) reagent (Promega, Madison, WI, USA) was added for 2 h to visualize the colonies. Colonies were counted using the ImageJ software package.

### Apoptotic cell death

For the evaluation of apoptotic cell death, cells were treated as indicated for the given time points. Cell death analysis was done using two different approaches.

#### Apoptotic cell death

First, apoptosis rates were evaluated as described by Nicoletti et al.^60^. After treatment cells were harvested, washed with ice cold PBS and pelleted (600×*g*, 5 min, 4 °C), before being incubated with hypotonic fluorochrome solution containing propidium iodide (HFS-PI solution). Cells were then analyzed via flow cytometry. Apoptotic cells are represented as a broad sub-G1 peak in the histogram plot and were gated accordingly.

#### Specific cell death

In the second approach, forward and side scatter plot signals (FSC-SSC) were used to determine the cell death of PDX cells and PBMCs. The FSC signal is a measure for cell size, while the SSC signal can be used to evaluate the granularity of the cells. Cells were harvested and analyzed via flow cytometry. Apoptotic cells are characterized by shrinkage (reduced FSC signal) and higher particle content (increased SSC signal). Viable and dead cells were gated accordingly in a FSC-SSC dot plot.

#### Data analysis and synergy

The percentage of specific cell death was calculated as described previously using the formula 100 × [experimental cell death (%) − spontaneous cell death (%)]/[100% − spontaneous cell death (%)]^19^. For the evaluation of synergy two separate methods were used. First, Bliss values (BV) were calculated as described previously with the formula [specific cell death combined treatment (E_AB_)/(specific cell death compound 1 (E_A_) + specific cell death compound 2 (E_B_) − (E_A_) × (E_B_))]^61^. Synergistic effects are indicated by BV > 1.05, additive effects with BV = 0.95 − 1.05 and antagonistic effects with BV < 0.95. Further, the Response Additivity approach was used, which consists in showing that a positive drug combination effect occurs when the combination effect (E_AB_) is greater than the expected additive effect (see dashed lines) given by the sum of the individual effects (E_A_ + E_B_)^62^.

### Mitochondrial stress test

HEK293T cells were seeded at a density of 1.4 × 10^4^ in DMEM assay medium (Agilent Technologies, Santa Clara, CA) supplemented with d-glucose (10 mM), pyruvate (1 mM) and glutamine (2 mM). For seeding of HEK293T cells, XF^e^96 microplates were coated with Poly-d-lysine hydrobromide (0.1 g/L) for 5 min and cells were grown for 24 h prior to 334 treatment (10 µM and 30 µM, 24 h). Jurkat, K562 and CEM cells were treated with 334 (10 µM and 30 µM) for 24 h and on the day of the assay immobilized on Cell-Tak-coated XF^e^96 microplates. In detail, microplates were coated with 22.4 µg/ml Cell-Tak solution (Corning; NY, USA) for 20 min and suspension cells (5.0 × 10^4^ cells/well) were seeded in RPMI assay medium (Agilent Technologies, Santa Clara, CA) supplemented with D-glucose (10 mM), pyruvate (1 mM) and glutamine (2 mM). For fixation of suspension cells, the microplate was centrifuged (200 g, zero braking) for 1 min, 130 µL assay medium was added to all wells and the plate was transferred to a 37 °C incubator not supplemented with CO_2_ for 1 h prior to the assay. Measurements were performed using the Agilent Seahorse XF Cell Mito Stress Test Kit in combination with the Seahorse XFe96 Analyzer (Agilent Technologies, Santa Clara, CA). Oligomycin (2.5 µM final concentration), FCCP and Rotenone/Antimycin A (0.5 µM final concentration) were injected into each well and the oxygen consumption rates (OCRs) was assessed as indicated**.** The optimal FCCP concentration was determined separately for all cell lines (see Supplementary Fig. S6 online). For HEK293T cells, the OCRs were normalized to the DNA content via staining with the CyQuant GR dye solution (Thermo Fisher Scientific, Waltham, MA) according to the manufactures protocol. Data analysis was performed using the Wave 2.6.1 software and the Seahorse XF Cell Mito Stress Test Report Generator (Agilent Technologies, Santa Clara, CA).

### Measurement of mitochrondrial ROS production

For the evaluation of reactive oxygen species (ROS) produced by mitochondria, MitoSOX dye (Thermo Fisher Scientific, Waltham, MA, USA) was used according to the manufacturer’s manual. In short, after an incubation with the indicated compounds for 6 h cells were harvested and washed with PBS. Before harvesting, cells of the positive control were pretreated with Antimycin A (20 μM, 45 min, 37 °C, 5% CO_2_), an inhibitor of the electron transport chain (ETC) complex III (Sigma-Aldrich, Taufkirchen, Germany). Thereafter all cells were incubated with MitoSOX working solution (5 µM in HBSS buffer) for 30 min (37 °C, 5% CO_2_), washed, resuspended in PBS and analyzed via flow cytometry. Mitochondrial ROS production was evaluated using mean fluorescence intensities (MFI) and histogram plots.

### Measurement of mitochondrial membrane potential (MMP)

Mitochondrial membrane potential (MMP) was analyzed using the cationic dye JC-1 (Enzo Life Sciences, Farmingdale, NY, USA). Monomeric JC-1 shows green fluorescence, while aggregates formed in mitochondria with intact membrane potential show red fluorescence. After cells were treated with respective compounds for the indicated time points, JC-1 staining solution was added directly to the wells for 30 min (37 °C, 5% CO_2_) for a final concentration of 2 µM. Carbonyl cyanide 3-chlorophenylhydrazone (CCCP) (Sigma-Aldrich, Taufkirchen, Germany), which causes a disruption of the MMP, served as a positive control and was added during the staining process (50 µM). Red and green fluorescence was analyzed using flow cytometry and cells with intact MMP were gated in dot plots. For signal compensation BD CompBeads Anti-Mouse Ig, κ particles and AlexaFluor488/PE mouse IgG2b, κ isotype control antibodies (BD Biosciences, Heidelberg, Germany) were used according to the manufacturer’s protocol.

### Migration— modified Boyden chamber assay

Migrational ability of cells was assessed using the boyden chamber assay. HUH7 cells were seeded in 6-well plates and either left untreated or pretreated with the indicated compound for 24 h. After pre-incubation, 1 × 10^5^ cells per condition were resuspended in growth medium containing the respective compounds and were transferred into collagen G coated Transwell Permeable Supports (8 µm pore polycarbonate inserts, Corning Inc., New York, NY). The transwell inserts were then placed into a 24-well plate containing 700 µL of DMEM (containing 10% FCS) and incubated for 16 h (37 °C, 5% CO_2_). Migrated cells were stained with crystal violet and counted using the particle counter plugin of the ImageJ software.

### Mitochondrial morphology

#### Fluorescence staining

Mitochondrial morphology was analyzed using MitoTracker Red CMXRos (Thermo Fisher Scientific, Waltham, MA, USA) according to the manufacturer’s instructions. For this purpose, MDA-MB-231 cells were seeded in 8-well ibiTreat μ-slides (ibidi GmbH, Munich, Germany) and treated with respective compounds as indicated. After the treatment cells were incubated with growth medium containing staining solution (200 nM) for 30 min (37 °C, 5% CO_2_), before mitochrondial morphology was evaluated via live-cell imaging with a Leica-SP8 confocal microscope (Leica Microsystems, Wetzlar, Germany) equipped with an incubation chamber (37 °C, 5% CO_2_, 80% humidity; okolab S.r.l., Pozzuoli, Italy).

#### Transmission electron microscopy (TEM)

Further, mitochondrial morphology was analyzed via electron microscopy. For this purpose, 0.5 × 10^6^ Jurkat cells were treated as indicated, washed in PBS, pelleted (410 g, 10 min, RT) and transferred into 0.95 ml BEEM capsules (Plano GmbH, Wetzlar, Germany). Cells were fixed in 6.25% glutaraldehyde in sodium cacodylate buffer (Electron Microscopy Sciences, Hatfield, PA, USA) for at least 24 h. Thereafter glutaraldehyde was removed and samples were washed three times with 0.1 M sodium cacodylate buffer, pH 7.4 (Electron Microscopy Sciences, Hatfield, PA, USA). Postfixation and prestaining was done for 45 to 60 min with 1% osmium tetroxide. Samples were washed three times with ddH_2_O and dehydrated with an ascending ethanol series (15 min with 30%, 50%, 70%, 90%, 96% ethanol, twice 10 min with 100%) and twice 30 min with propylene oxide (Serva Electrophoresis GmbH, Heidelberg, Germany). Subsequently, samples were embedded in Epon (3.61 M Glycidether 100, 1.83 M Methylnadicanhydride, 0.92 M Dodecenylsuccinic anhydride, 5.53 mM 2,4,6-Tris-(dimethylaminomethyl)phenol (Serva Electrophoresis GmbH, Heidelberg, Germany)). Ultrathin sections were sliced with an Ultramicrotome (Ultracut E; Reichert und Jung, Germany) and stained with UranyLess EM Stain (Electron Microscopy Sciences) and 3% lead citrate (Leica, Wetzlar, Germany) using the contrasting system Leica EM AC20 (Leica, Wetzlar, Germany). Pictures were taken with a JEOL-1200 EXII transmission electron microscope (JEOL GmbH, Freising, Germany).

### Crystal violet staining

Crystal violet staining was used for measurements of proliferation and cell number. Therefore, cells were seeded in triplicates (10.000 cells/well) in 96 well plates and grown for 24 h. Cells were treated with the indicated compounds (72 h), stained with crystal violet solution (0.5% crystal violet, 20% Methanol, in H_2_O) for 10 min at RT and washed with distilled H_2_O to removed excessive staining solution. The bound dye was solubilized with dissolving buffer (50 mM Trisodiumcitrate, 50% Ethanol, in H_2_O) and the proliferation was quantified by absorbance measurement at 550 nm with a SpectraFluorPLUS plate-reading photometer.

### Quantification of protein carbonylation

For analysis of protein carbonylation the Oxidized Protein Western Blot Kit (Abcam, Cambridge, UK) was used according to the manufacturer’s protocol. In brief, Jurkat cells were treated with 334 or DMSO for 6 h. H_2_O_2_ pretreatment (1 mM, 1 h) was used as a positive control. Cells were lysed and carbonyl groups in the protein side chains were derivatized to 2,4-dinitrophenylhydrazone (DNP-hydrazone) by incubation with 2,4-dinitrophenylhydrazine (DNPH). SDS-PAGE and immunoblotting against DNP were performed as described above. The DNP abundance was quantifies by normalization to the respective protein load.

### Quantitative real-time PCR analysis

For the mRNA isolation from cell culture samples the Qiagen RNeasy Mini Kit (Qiagen, Hilden, Germany) was used according to the manufacturer’s protocol. The concentration of mRNA in each sample was determined with the NanoDrop ND 1000 spectrophotomer (Peqlab Biotechnology GmbH, Erlangen, Germany). For the creation of cDNA templates the High-Capacity cDNA Reverse Transcription Kit (Applied Biosystems, Foster City, CA) was used as described by the manufacturer. The Real-Time-Polymerase chain reaction (RT-PCR) was performed with the QuantStudio 3 Real-Time PCR System (Applied Biosystems, Foster City, CA). SYBR Green Mix I (Applied Biosystems, Foster City, CA, USA) was used for the RT-PCR of CHOP (forward primer: 5′-TTG CCT TTC TCC TTC GGG AC-3′, reverse primer: 5′-CAG TCA GCC AAG CCA GAG AA -3′) and Hsp60 (forward primer: 5′-GGA CAC GGG CTC ATT GCG-3′, reverse primer: 5′-TTC TTC AGG GGT GGT CAC AG-3′). Actin was used as a housekeeping gene (forward primer: 5′-TTC ACC TAC AGC AAG GAC GA-3′, reverse primer: 5′-GAA CTC GAA GAT GGG GTT GA-3′) and average CT values of target genes were normalized to the housekeeper control as ΔCT. Changes in RNA levels were shown as x-fold expression (2−ΔΔ CT) calculated by the ΔΔCT method^63^.

### Statistics

All experiments described were conducted at least three times. The data are presented as the mean ± SEM, and statistical significance was considered at P ≤ 0.05. Statistical analysis was performed with GraphPad Prism software 7.0. For differences between two groups, an unpaired two-tailed Student’s t-test was used. Group comparisons were performed using one-way ANOVA with Tukey’s multiple comparison test.

## Supplementary Information

Supplementary Information.

## Data Availability

All data generated or analyzed during this study are included in the manuscript and supporting files.

## Abbreviations

AML: Acute myeloid leukemia
CDDO-ME: Bardoxolone methyl
CHOP: C/EBP homologous protein
CML: Chronic myeloid leukemia
ER: Endoplasmatic reticulum
ETC: Electron transport chain
HCC: Hepatocellular carcinoma
HsClpXP: Human ClpXP
HspX: Heat shock protein X
HTS: High-throughput screen
MMP: Mitochondrial membrane potential
mtUPR: Mitochondrial unfolded protein response
MS: Mass spectrometry
OCR: Oxygen consumption rate
PARP: Poly [ADP-ribose] polymerase
PBMC: Peripheral blood mononuclear cell
PDX: Patient-derived xenograft
ROS: Reactive oxygen species
SaClpXP: *Staphylococcus aureus* (*S. aureus*) ClpXP
SILAC: Stable isotope labeling with amino acids in cell culture
T-ALL: T-cell acute lymphoblastic leukemia
TEM: Transmission electron microscopy
VCR: Vincristine-resistant
