# Supplementary material for: Small molecule inhibitors of the mitochondrial ClpXP protease possess cytostatic potential and re-sensitize chemo-resistant cancers
Source: Sci Rep. 2021 May 27;11:11185. doi: 10.1038/s41598-021-90801-7 (PMC8160014; doi:10.1038/s41598-021-90801-7)

Supplementary information

**Title:**

Small molecule inhibitors of the mitochondrial ClpXP protease possess cytostatic potential and re-sensitize chemo-resistant

**Authors:**

Martina Meßner^1,2†^ & Melanie M. Mandl^1†^; Mathias W. Hackl^3^; Till Reinhardt^3^, Maximilian A. Ardelt^2^; Karolina Szczepanowska^4^; Julian E. Frädrich^1^; Jens Waschke^5^; Irmela Jeremias^6,7^; Anja Fux^3^; Matthias Stahl^3,8^; Angelika M. Vollmar^1^; Stephan A. Sieber^3^* & Johanna Pachmayr^2,9^*

**Affiliations:**

^1^Department of Pharmacy, Pharmaceutical Biology, Ludwig-Maximilians-University (LMU) Munich, Munich, 81377, Germany

^2^Paracelsus Medical University, Institute of Pharmacy, Salzburg, 5020, Austria

^3^Center for Integrated Protein Science Munich, Department of Chemistry, Technical University Munich, 85748 Garching, Germany

^4^Institute for Mitochondrial Diseases and Aging at CECAD Research Centre, and Center for Molecular Medicine Cologne (CMMC), Medical Faculty, University of Cologne, Cologne, Germany

^5^Faculty of Medicine, Institute of Anatomy, Ludwig-Maximilians-University (LMU) Munich, 80336, Munich, Germany

^6^Research Unit Apoptosis in Hematopoietic Stem Cells, Helmholtz Zentrum München, Munich, 81377, Germany

^7^Dr. von Hauner Children's Hospital, Ludwig Maximilians University (LMU), Munich, 80337, Germany

^8^Present address: Science for Life Laboratory, Department of Oncology-Pathology, Karolinska Institutet, Box 1031, 171 21 Solna, Stockholm, Sweden

^9^Paracelsus Medical University, Institute of Pharmacy, Center for Public Health, Salzburg, 5020, Austria

*Corresponding authors: Prof. Johanna Pachmayr & Prof. Stephan A. Sieber

†M.M. and M.M.M. contributed equally

**Overview:**

**Supplementary Figures:**

Supplementary Figure 1 │ SaClpXP inhibitors that were tested for activity against the human ClpXP.

Supplementary Figure 2 │ 334 treatment impairs cell proliferation of HCC and breast cancer cells.

Supplementary Figure 3 │ 339 treatment prevents migration of HCC cells at non-lethal concentrations.

Supplementary Figure 4 │ MS-based proteomics screen of K562 cells upon 24 h and 48 h of 334 treatment.

Supplementary Figure 5 │ 334 promotes cellular stress response and slightly affects mitochondrial localization.

Supplementary Figure 6 │ Parameter calculation and FCCP titration for mitochondrial stress test.

Supplementary Figure 7 │ Chemo-sensitization by 334 not determined by ClpX protein expression.

Supplementary Figure 8 │ Uncropped Western blots of main figures.

**Supplementary Tables:**

Supplementary Table 1 │ Clinical data of patients donating diagnostic AML and ALL cells for xenotransplantation.

Supplementary Table 2 │ Depleted proteins identified by quantitative MS upon 24 h of 334 treatment (10 µM).

Supplementary Table 3 │ Enriched proteins identified by quantitative MS upon 24 h of 334 treatment (10 µM).

Supplementary Table 4 │ Depleted proteins identified by quantitative MS upon 48 h of 334 treatment (10 µM).

Supplementary Table 5 │ Enriched proteins identified by quantitative MS upon 48 h of 334 treatment (10 µM).

**Supplementary Figures**


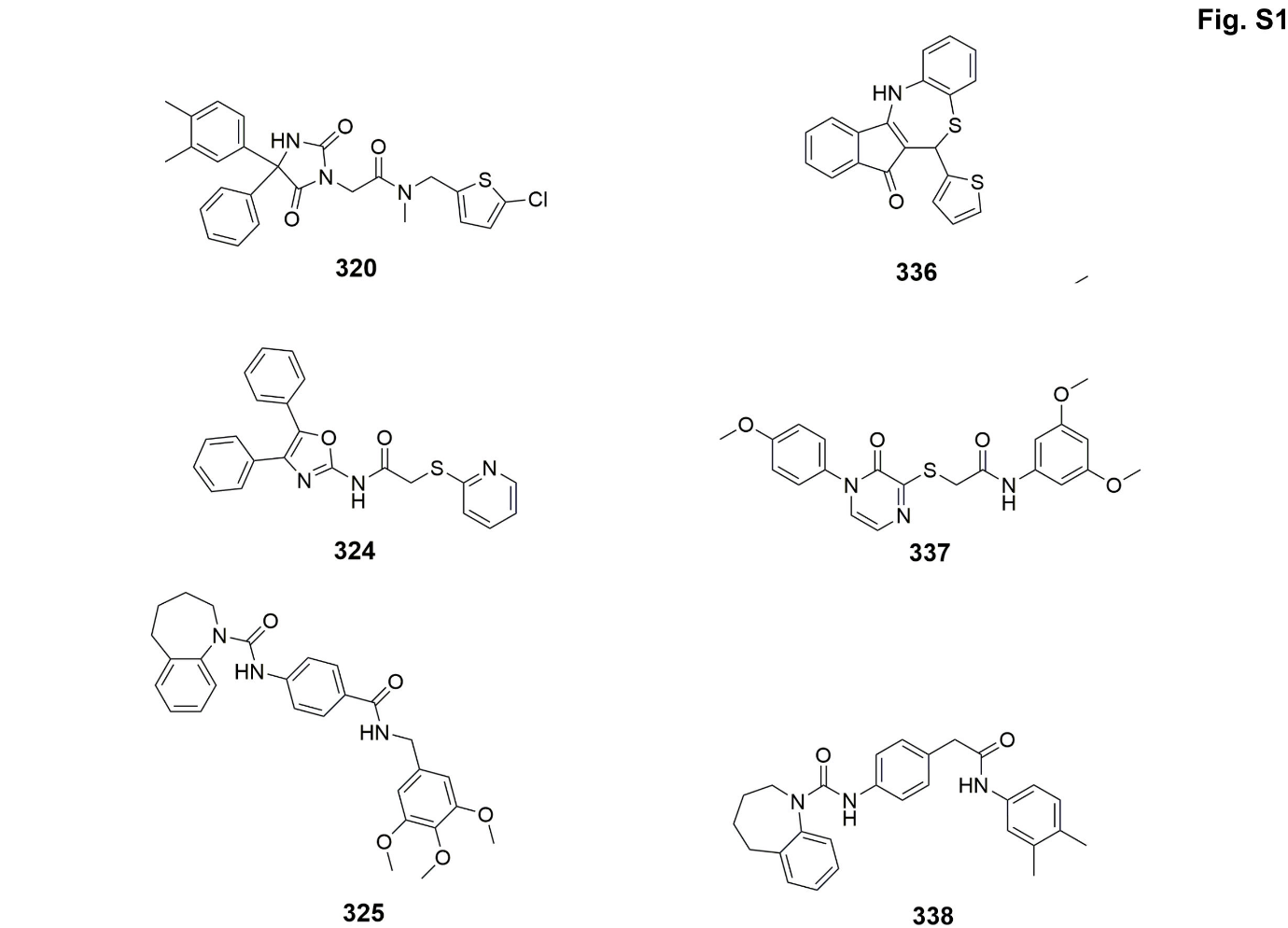


**Fig. S1: SaClpXP inhibitors that were tested for activity against the human ClpXP**.

The chemical structures of the SaClpXP small molecule inhibitors tested in Figure *Fig. 1b* are shown. The structures of the small molecules 319, 334 and 339 are presented in *Fig.1a*.


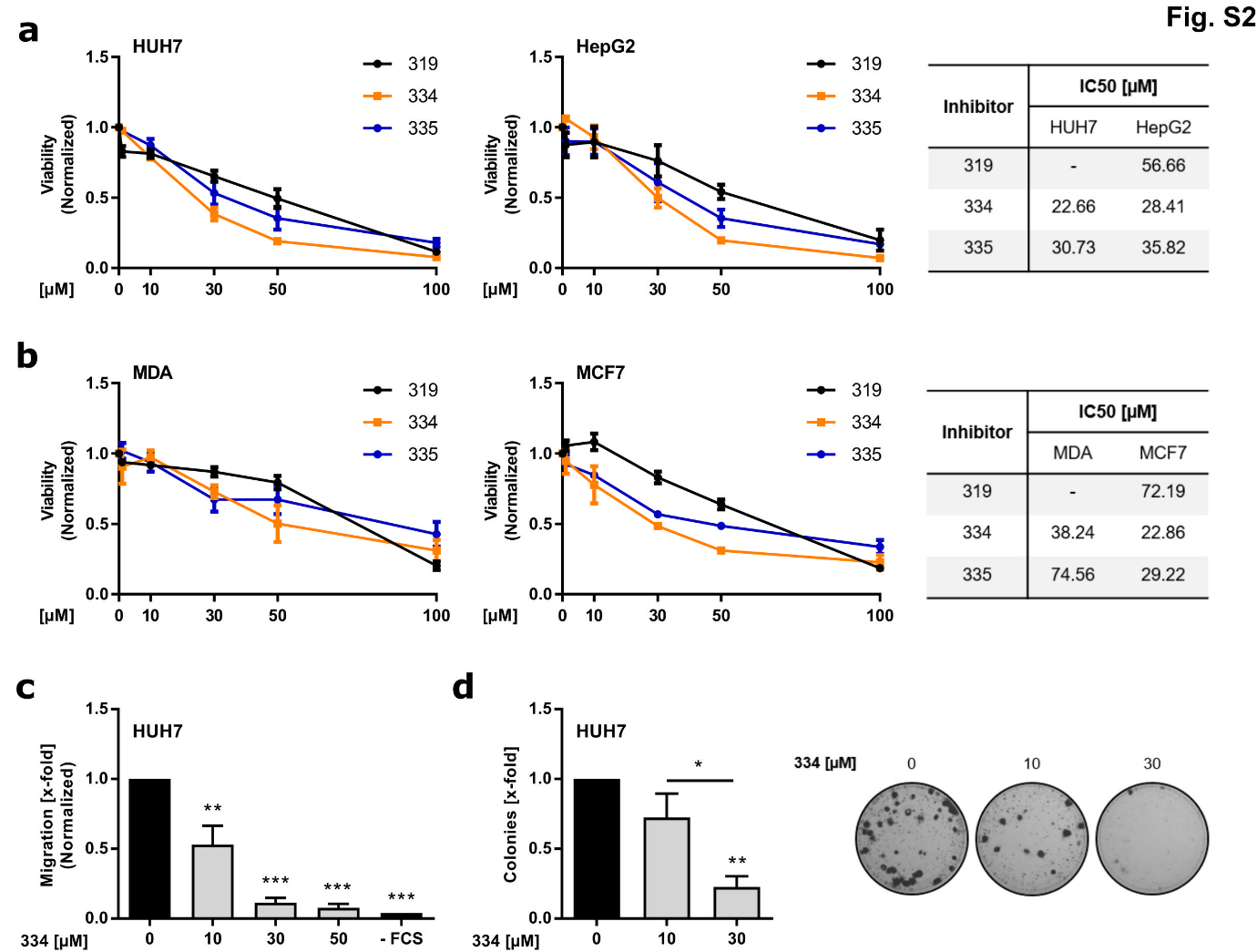


**Fig. S2: 334 treatment impairs cell proliferation of HCC and breast cancer cells**.

1. HCC cells show strongest inhibition of proliferation upon 334 treatment. Cell viability of HUH7 and HepG2 HCC cell lines upon 319, 334 or 335 treatment for 72 h was normalized to the DMSO treated control. Corresponding IC_50_ values were calculated by nonlinear regression analysis (n=3).
2. The compounds 334 and 335 inhibit proliferation of breast cancer cells. Cell viability of MDA and MCF7 breast cancer cell lines upon 319, 334 or 335 treatment for 72 h was normalized to the DMSO treated control. Corresponding IC_50_ values were calculated by nonlinear regression analysis (n=3).
3. 334 treatment for 16 h impairs HCC cell migration. Migration was assessed by the Boyden chamber assay. Migrated cells were stained with crystal violet, counted and normalized to the untreated control. A chamber without FCS in the outer compartment serves as negative control (- FCS) (***P*<0.01, ****P*<0.001, One-way ANOVA, Tukey’s Multiple Comparison Test, n=3).
4. 334 treatment impairs clonogenic growth of HCC cells. Colony count of HUH7 cells normalized to the untreated control is shown (left panels). Representative images of colonies formed by HUH7 cells pretreated with 334 for 24 h and grown in the absence of 334 for further 7 days are shown (right panel) (**P*<0.05, ***P*<0.01, One-way ANOVA, Tukey’s Multiple Comparison Test, n=3).


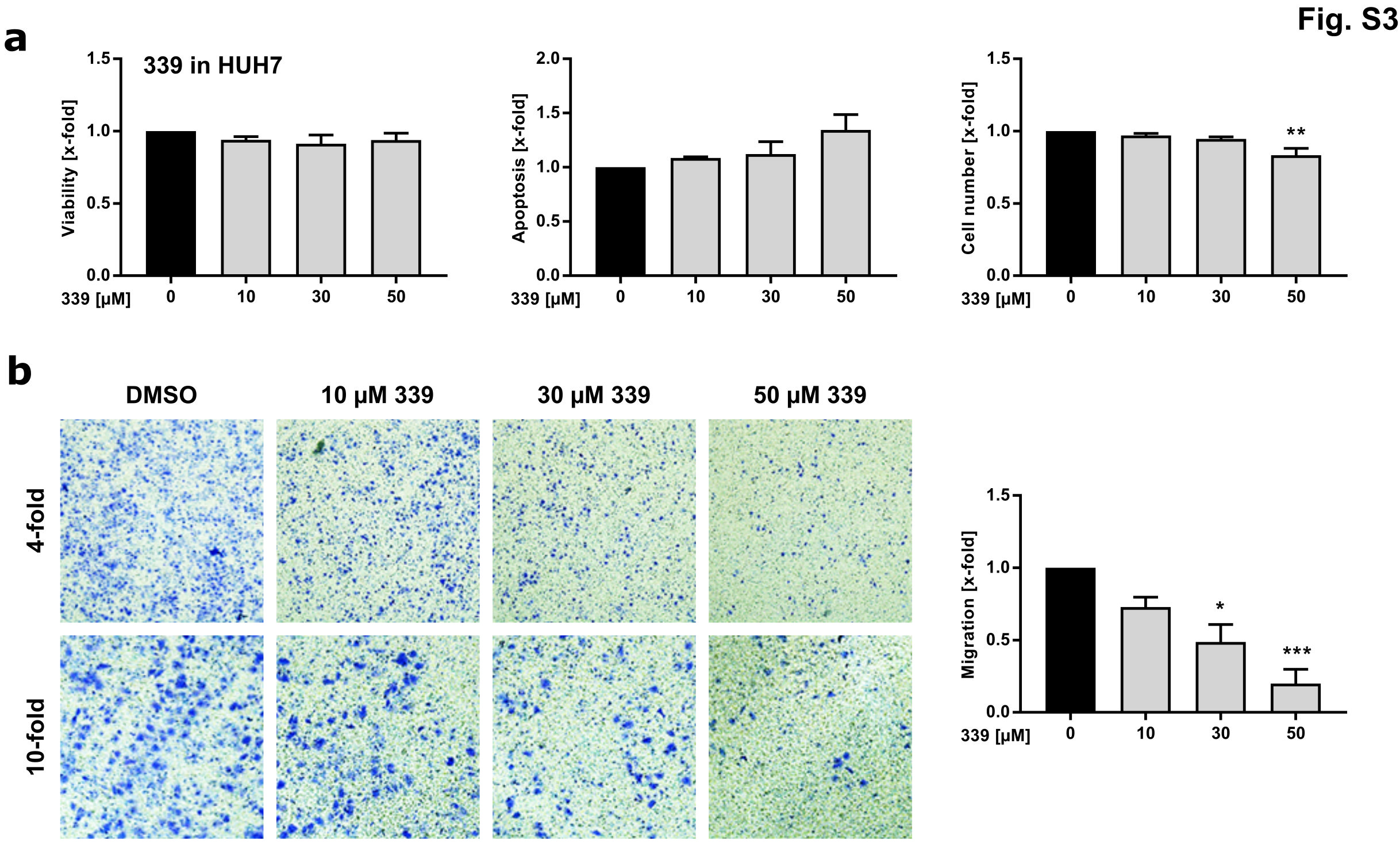


**Fig. S3: 339 treatment prevents migration of HCC cells at non-lethal concentrations**.

1. HCC cell proliferation is not impaired by 339. HUH7 cells were treated with 339 and cell viability (3 h), apoptosis (24 h) respectively cell number (72 h) were assessed as x-fold change of the untreated control (***P*<0.01, One-way ANOVA, Tukey’s Multiple Comparison Test, n=3).
2. 339 treatment for 16 h impairs HCC cell migration. Migration of HUH7 cells was assessed by the Boyden chamber assay. Migrated cells were stained with crystal violet, counted and normalized to the DMSO treated control (**P*<0.05, ****P*<0.001, One-way ANOVA, Tukey’s Multiple Comparison Test, n=3).


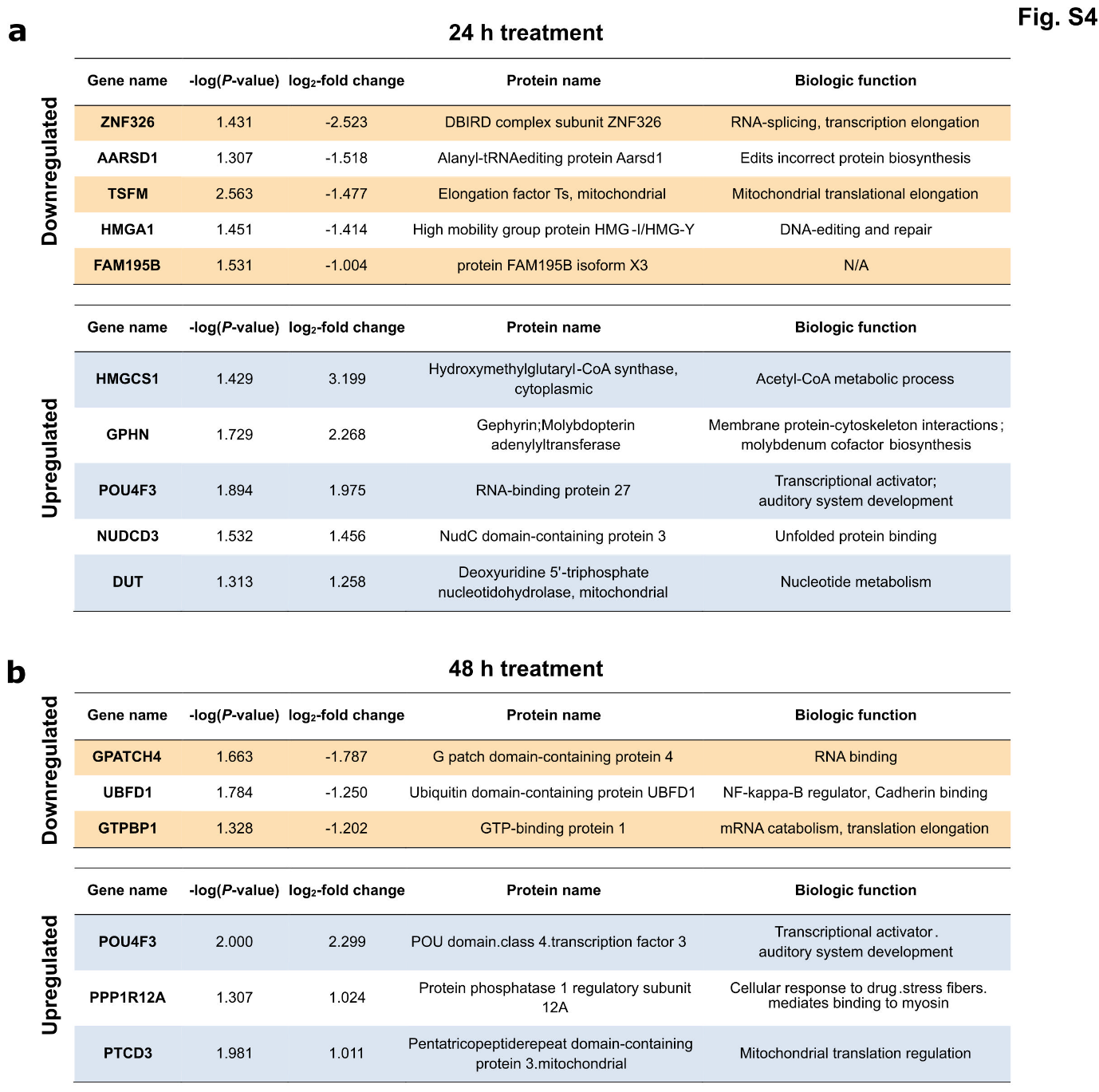


**Fig. S4: MS-based proteomics screen of K562 cells upon 24 h and 48 h of 334 treatment**.

Significantly depleted and enriched proteins (*P*< 0.05; |log_2_(334 [10 µM]/DMSO)| > 1) identified by quantitative mass spectrometry comparing 334-treated (10 µM) to DMSO-treated K562 cells upon

1. 24 h of treatment (Figure is related to Figure*Fig. 4a*)
2. and 48 h of treatment (Figure is related to Figure *Fig. 4b*).


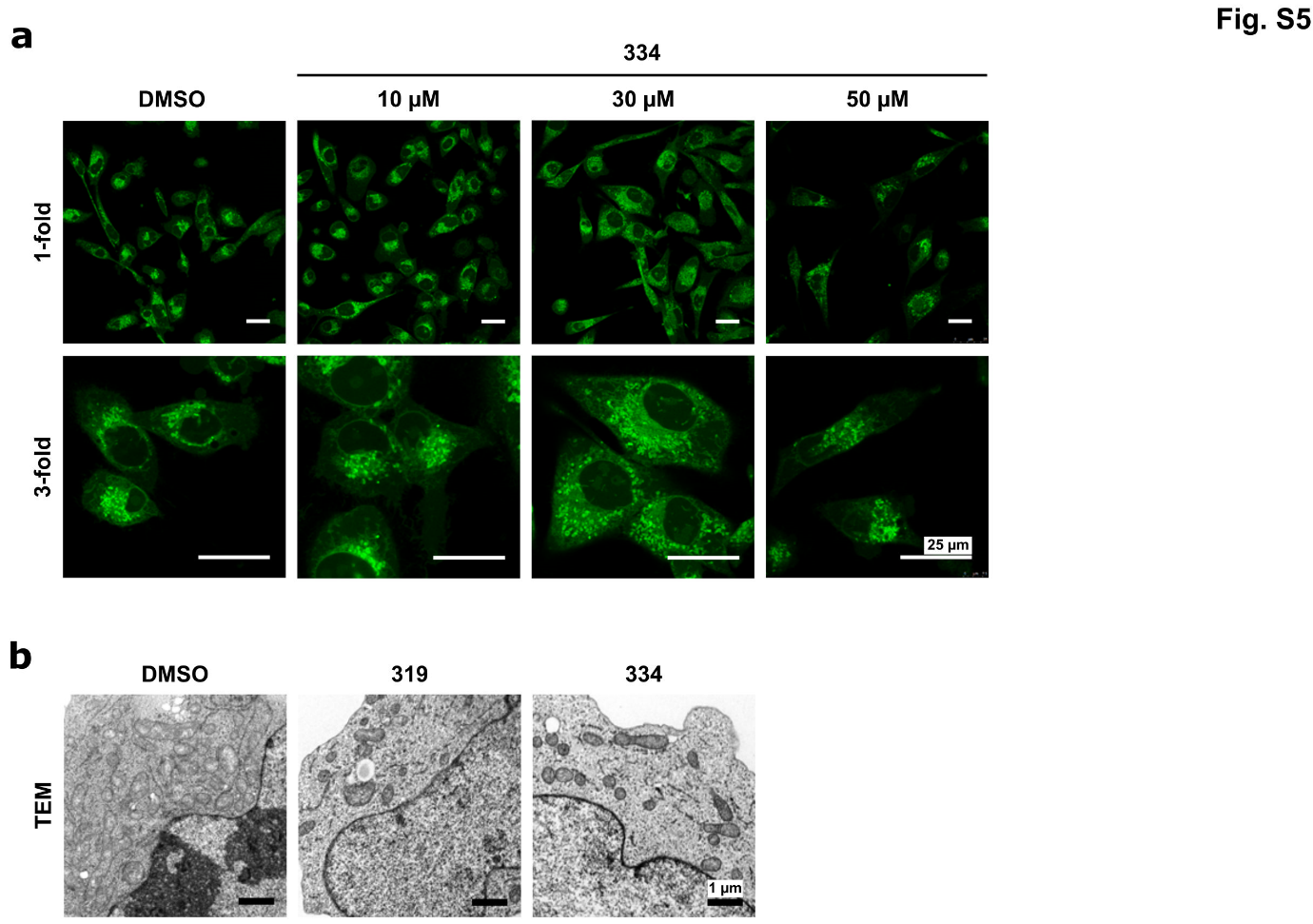


**Fig. S5: 334 promotes cellular stress response and slightly affects mitochondrial localization.**

1. 334 treatment promotes diffuse mitochondrial appearance. Confocal microscopy of living MDA-MB-231 cells stained with the MitoTracker Red CMXRos dye visualizes the mitochondrial network. Cells were either treated with DMSO (control) or 334 for 24 h. Scale bars indicate 25 µm (n=3).
2. 319 and 334 slightly affect mitochondrial mass and localization. Transmission electron microscopy (TEM) displays Jurkat cells treated with 319 (20 µM), 334 (20 µM) or DMSO for 24 h (n=3).

**
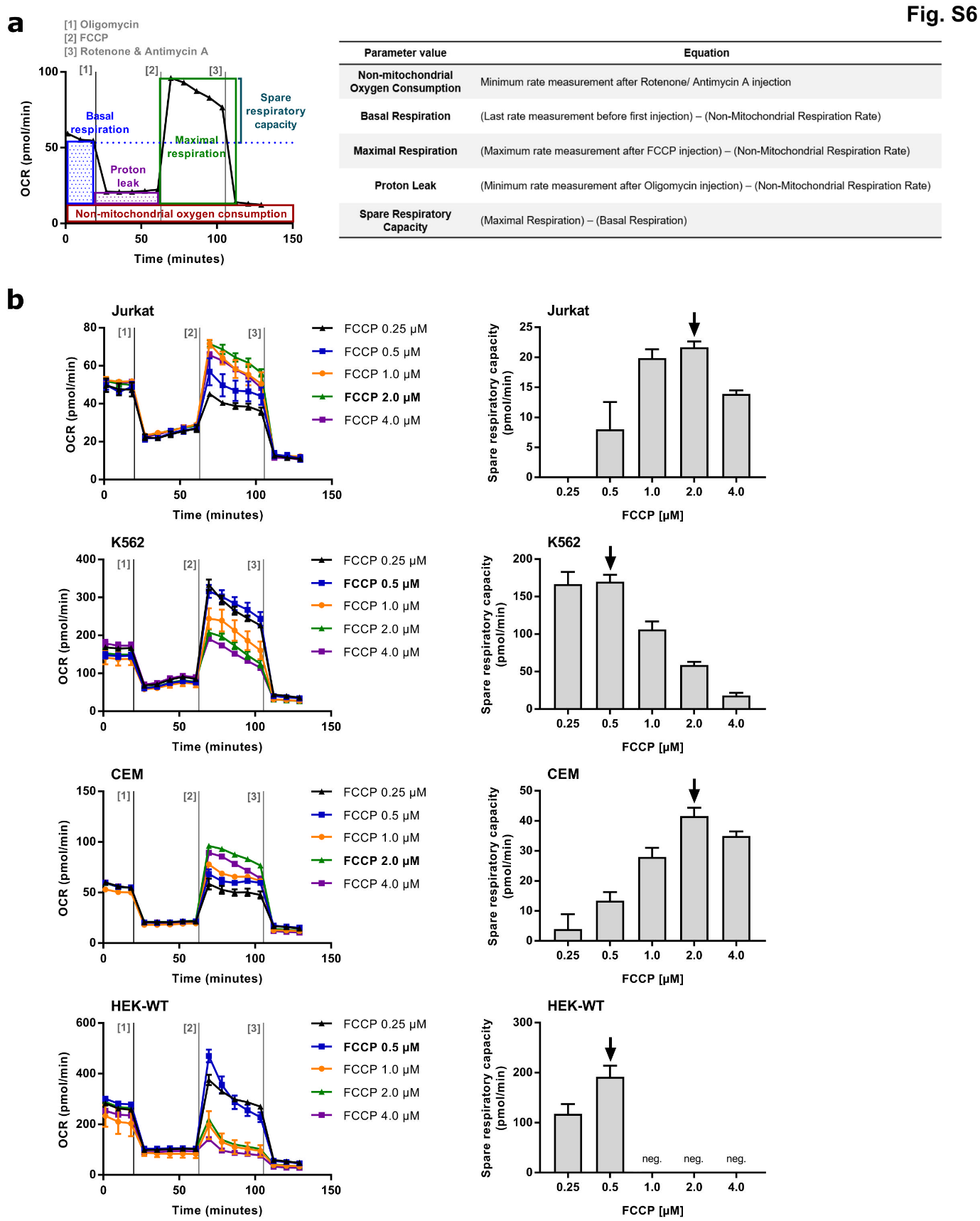
**

**Fig. S6: Parameter calculation and FCCP titration for mitochondrial stress test.**

1. Overview of parameter values, which were assessed by mitochondrial stress test on the Agilent Seahorse XF Analyzer (*Figure 5f*, *Figure 6c*, *Supplementary Figure S6b online*) and the equations, which were used for calculation of the respective values.
2. FCCP Titration of Jurkat, K562, CEM and HEK-WT cells. The optimal final FCCP concentration for achieving maximal OCRs is cell line dependent and is therefore determined by a FCCP titration experiment (0.25 µM, 0.5 µM, 1.0 µM, 2.0 µM, 4.0 µM). For FCCP titration, a constant oligomycin (2.5 µM) and Rotenone/ Antimycin A (0.5 µM) concentration is used as recommended by the manufacturer. Arrows (right panels) indicate the maximal spare respiratory capacity of each cell type and, thus, the FCCP concentration, which is used for further experiments (4 wells per condition, n=1).


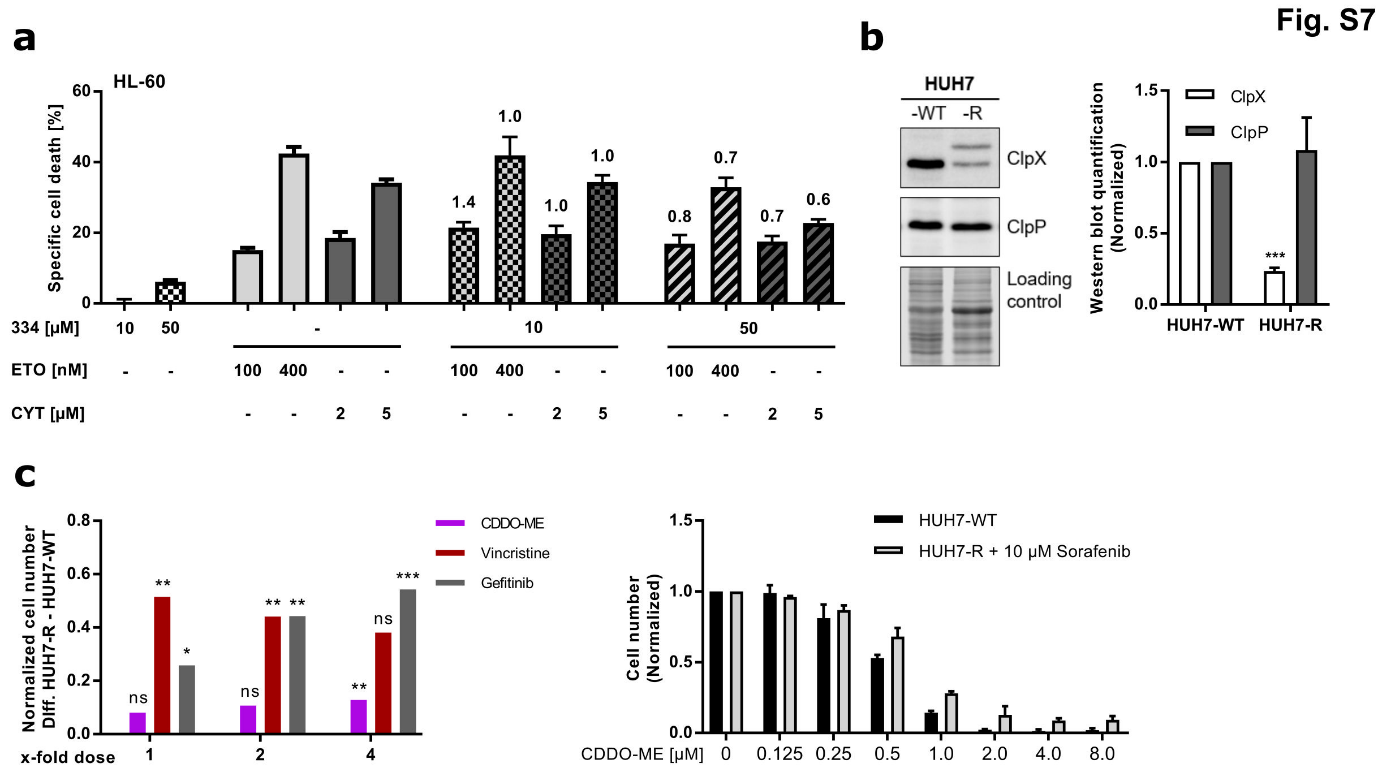


**Fig. S7: Chemo-sensitization by 334 not determined by ClpX protein expression.**

1. HL-60 cells were not sensitized to anticancer therapy by low-dose 334 combination treatment. Specific cell death induced in HL-60 cells upon 334 (10 µM), etoposide (ETO) and cytarabine (CYT) treatment is shown. Cells were treated with the indicated agents alone or in combination for 48 h. Cell death was assessed by flow cytometry using Nicoletti assay. Bliss values were calculated for combined chemotherapies and values above 1.05 indicate synergism (n=3).
2. HUH7-R are sensitized by 334 towards sorafenib therapy besides low ClpX protein expression. Western blot analysis was performed for ClpX and ClpP in HUH7-WT and HUH7-R cells. ClpX and ClpP protein expression was quantified and normalized to HUH7-WT and the respective protein load (****P*<0.001, t-test comparing cell types, n=3).
3. CDDO-ME re-sensitizes HUH7-R cells towards sorafenib therapy comparable to 334. Proliferation rates of HUH7-WT and HUH7-R cells within 72 h of treatment were assessed by crystal violet staining and normalized to the untreated control (right panel). Proliferation rates of vincristine and gefitinib treatment were previously shown (Meßner et al., 2020). The difference in growth rates obtained by HUH-R and HUH-WT cells was calculated and compared among treatments (left panel, see also *Figure 7b*) (*P<0.05, **P<0.01, ***P<0.001, One-way ANOVA, Tukey’s Multiple Comparison Test comparing concentrations, t-test comparing cell types, n=3).


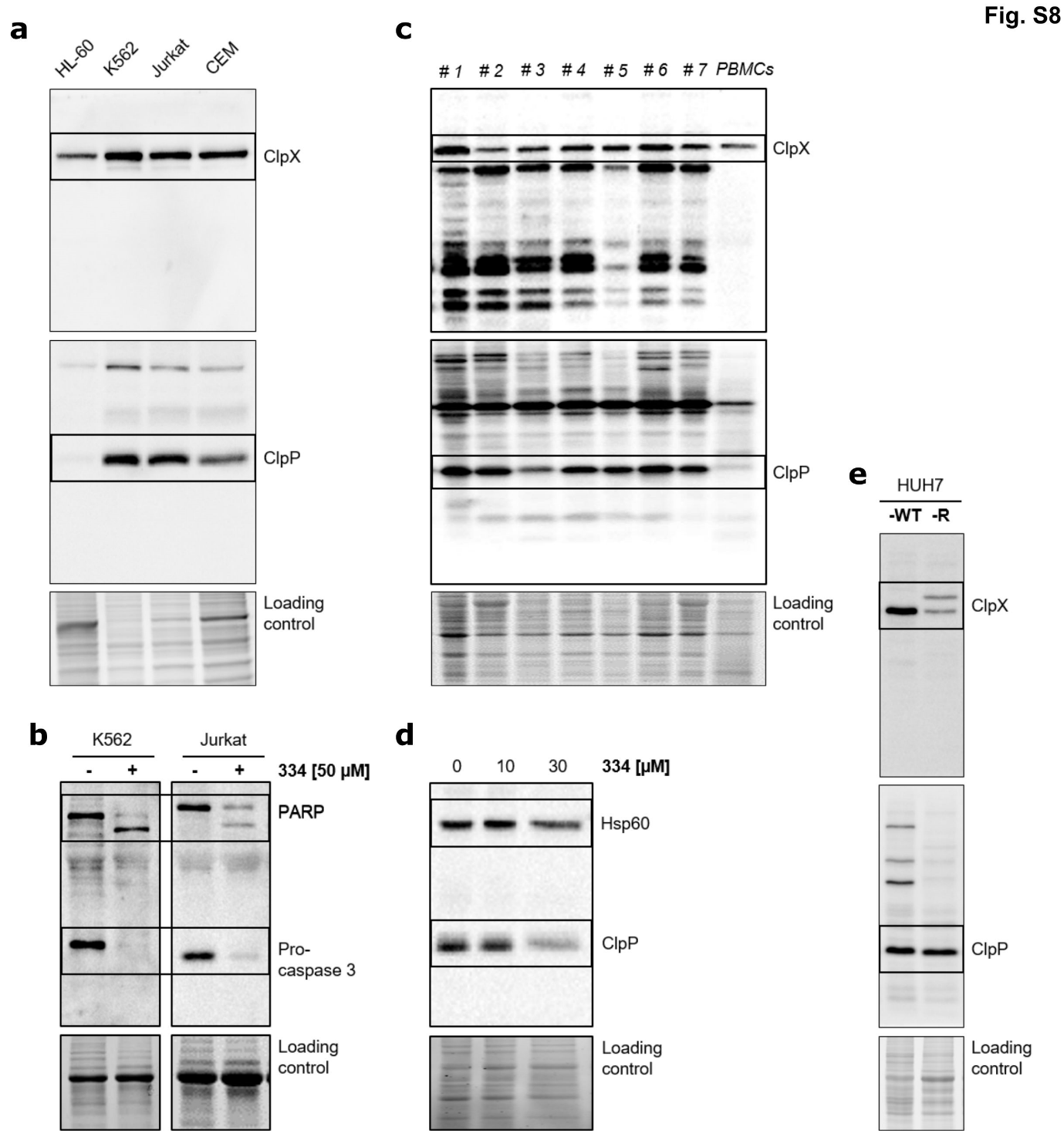


**Fig. S8: Uncropped Western blots of main figures.**

1. *Supplementary Figure S8a* is related to *Figure 2a* (right panel).
2. *Supplementary Figure S8b* is related to *Figure 2f* (upper right panel).
3. *Supplementary Figure S8c* is related to *Figure 3c*.
4. *Supplementary Figure S8d* is related to *Figure 4d*.
5. *Supplementary Figure S8e* is related to *Supplementary Figure S7b*.

**Supplementary Tables**

**Table. S1:** Clinical data of patients donating diagnostic AML and ALL cells for xenotransplantation. *Table is* related to Figure*Fig. 3.*


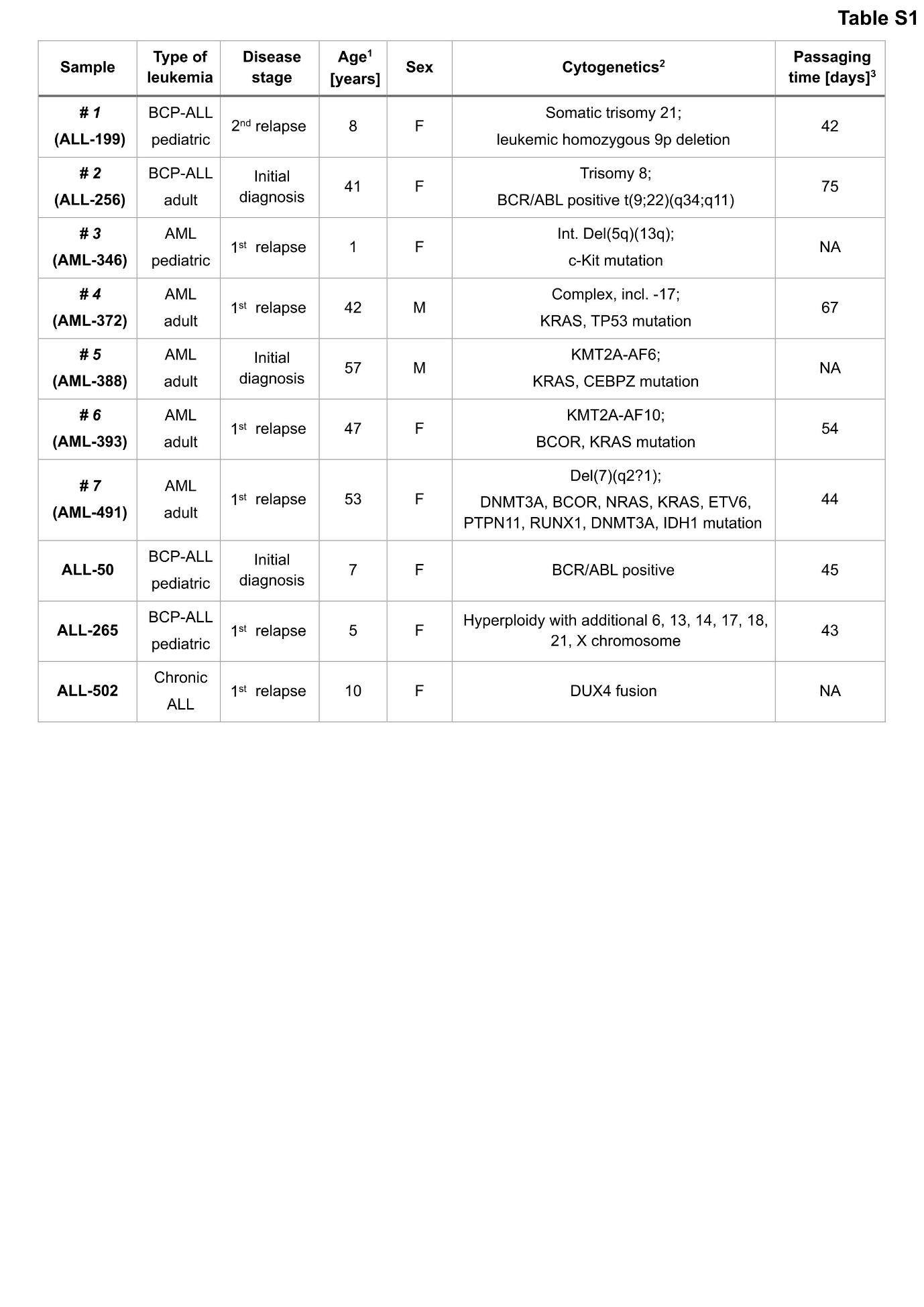


^1^when the primary AML or ALL sample was obtained; ^2^mutations detected by targeted re-sequencing in PDX cells; BCP, B cell precursor; ^3^time of passaging through mice refers to the time of sample injection until mice had to be sacrificed due to end stage leukemia; del, deletion; F, female; int., interstitial; M, male; NA, not available.

**Table. S2: Depleted proteins identified by quantitative MS** **upon 24 h of 334 treatment (10 µM).** The MS-proteomics screen compares 334-treated to DMSO-treated K562 cells. Proteins are ranked according to their x-fold change and proteins with a x-fold change ≤ -2 are shown. Supplementary *Table S2* and Supplementary *Table S3* are related to Figure*Fig. 4a* and *Supplementary Fig. S4a online*.


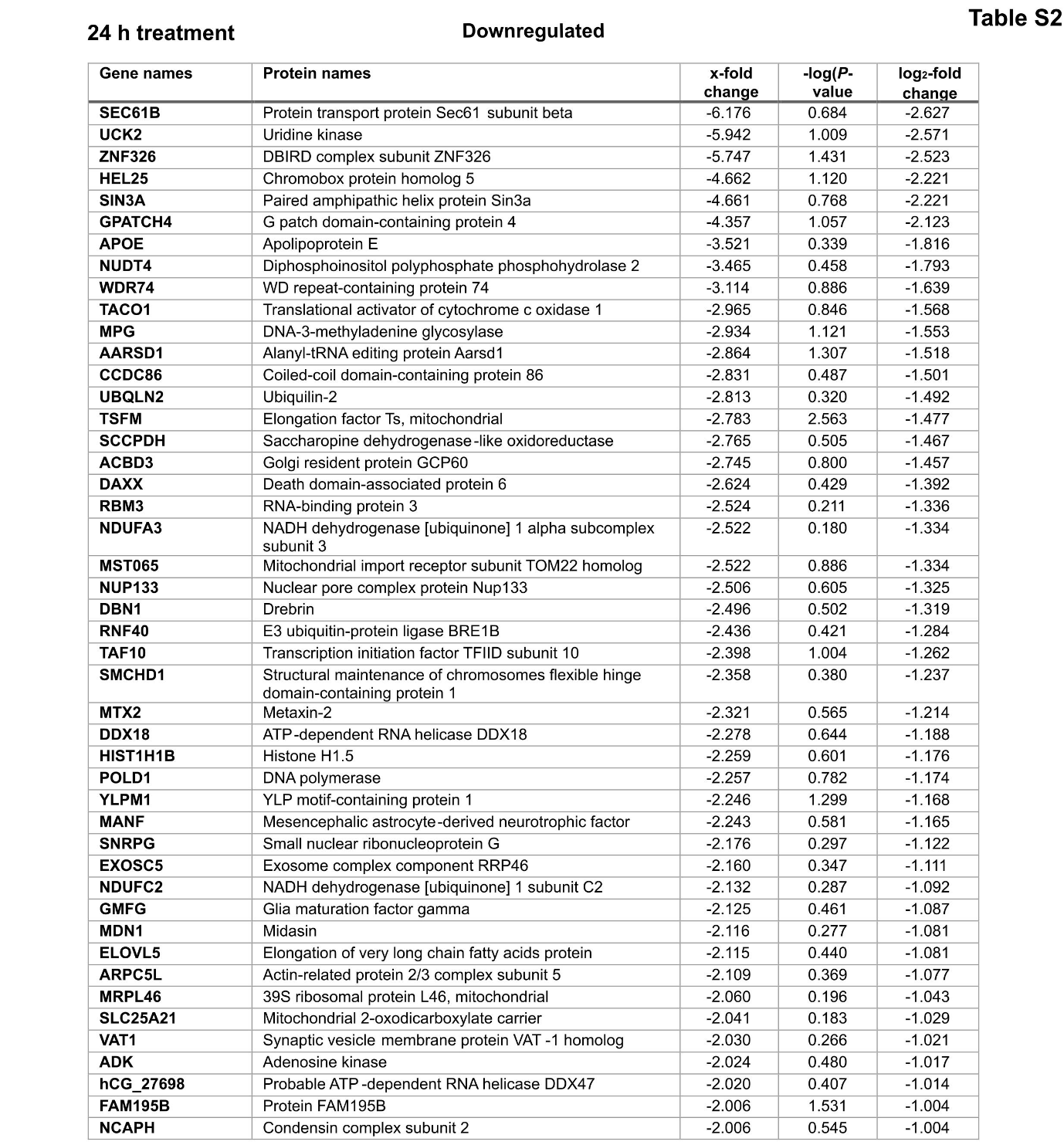


**Table. S3: Enriched proteins identified by quantitative MS** **upon 24 h of 334 treatment (10 µM).** Proteins are ranked according to their x-fold change and proteins with a x-fold change ≥ 2 are shown.


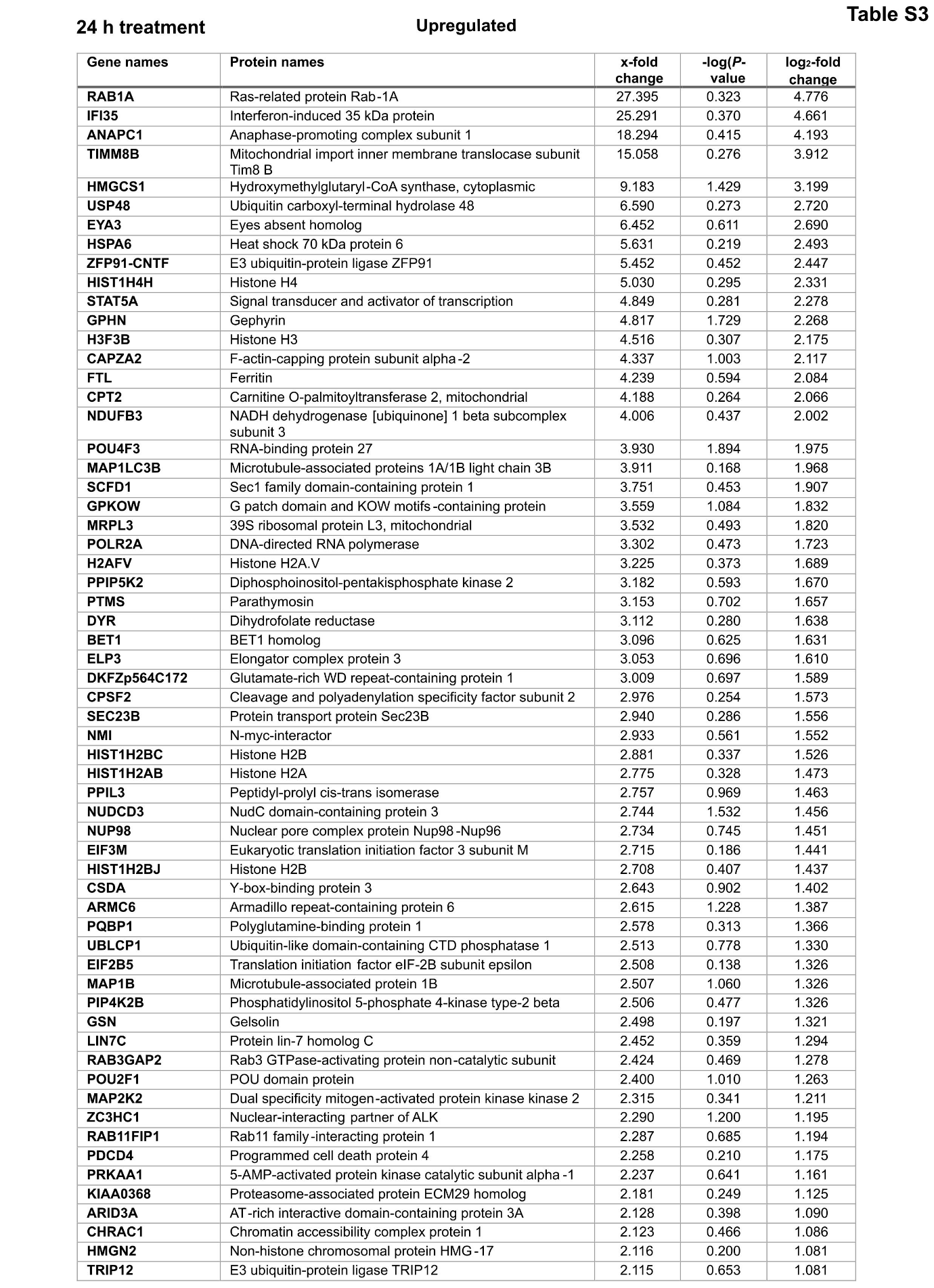


**Table. S4: Depleted proteins identified by quantitative MS upon 48 h of 334 treatment (10 µM).** The MS-proteomics screen compares 334-treated to DMSO-treated K562 cells. Proteins are ranked according to their x-fold change and proteins with a x-fold change ≤ -2 are shown. *Supplementary Table S4* and *Supplementary Table S5* are related to Figure *Fig. 4b* and Supplementary *Fig. S4b online*.


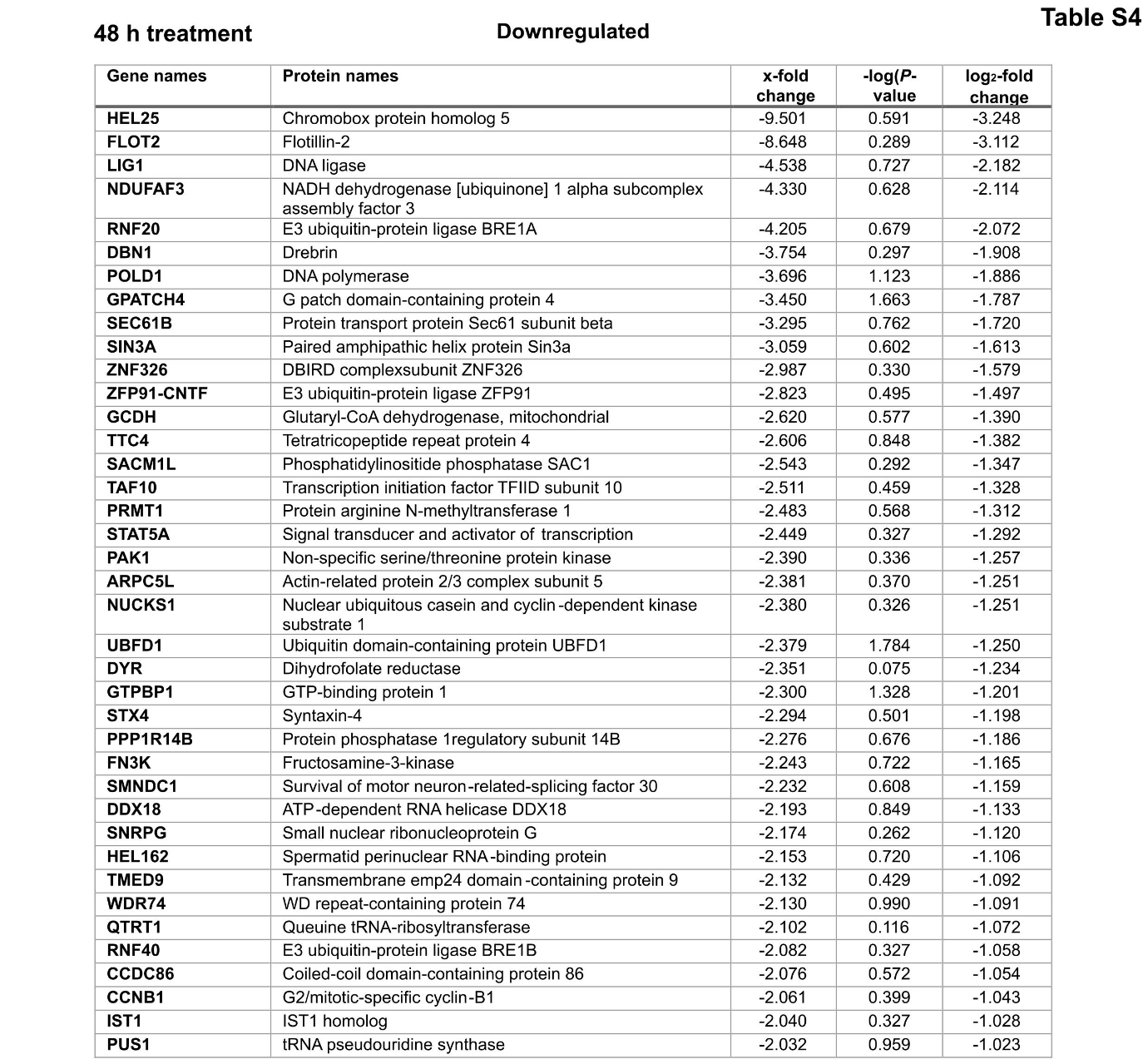


**Table. S5: Enriched proteins identified by quantitative MS** **upon 48 h of 334 treatment (10 µM).** Proteins are ranked according to their x-fold change and proteins with a x-fold change ≥ 2 are shown.


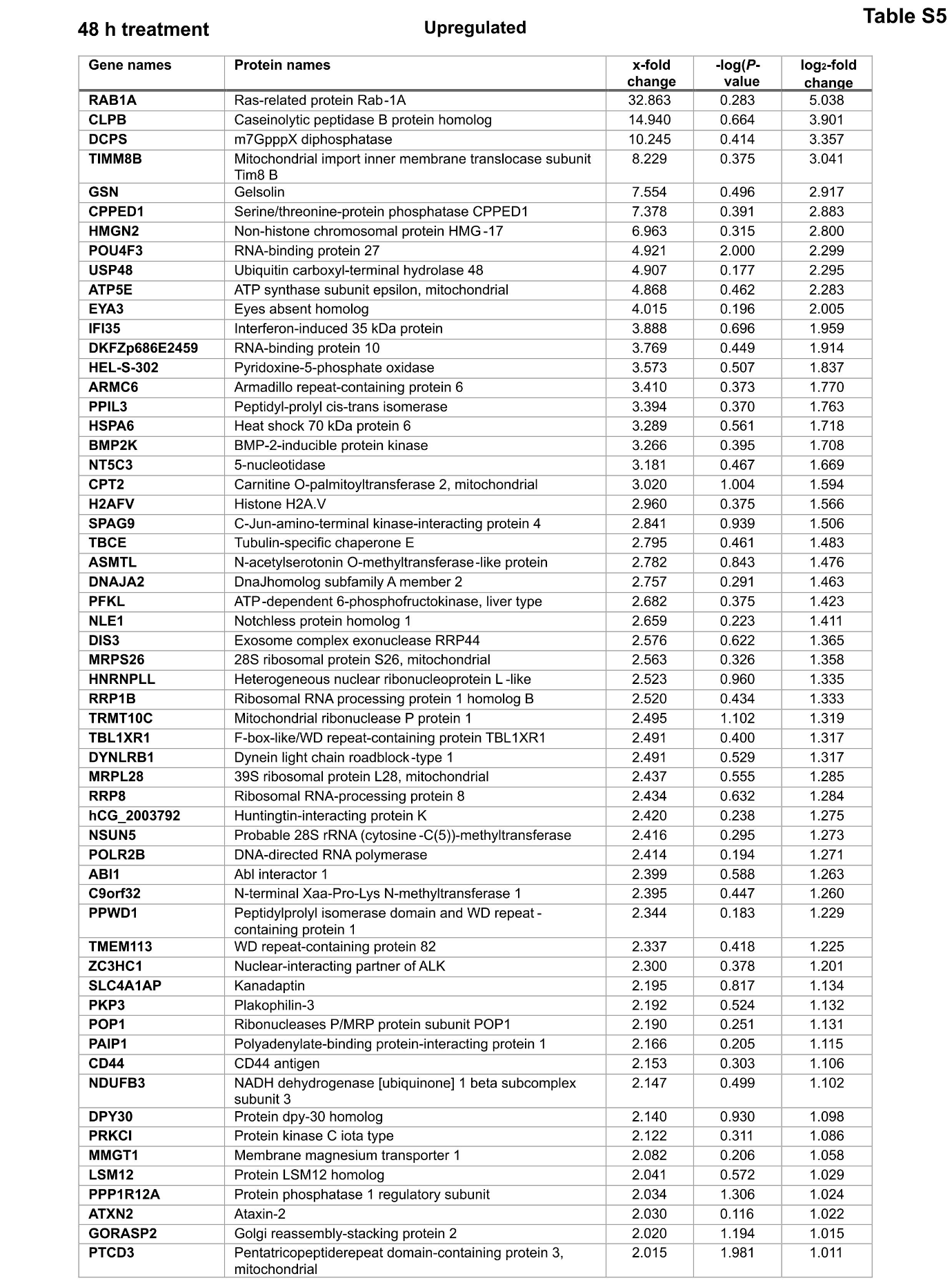

Supplement: Supplementary file 1 — Supplementary Information. [file 41598_2021_90801_MOESM1_ESM.docx]
